# Neural Tissue Homeostasis and Repair Is Regulated via CS and DS Proteoglycan Motifs

**DOI:** 10.3389/fcell.2021.696640

**Published:** 2021-08-02

**Authors:** Anthony J. Hayes, James Melrose

**Affiliations:** ^1^Bioimaging Research Hub, Cardiff School of Biosciences, Cardiff University, Wales, United Kingdom; ^2^Graduate School of Biomedical Engineering, University of New South Wales, Sydney, NSW, Australia; ^3^Raymond Purves Bone and Joint Research Laboratories, Kolling Institute of Medical Research, Royal North Shore Hospital and The Faculty of Medicine and Health, The University of Sydney, St. Leonard’s, NSW, Australia

**Keywords:** chondroitin sulfate, dermatan sulfate, proteoglycans, lecticans, neuroregulation, neural repair

## Abstract

Chondroitin sulfate (CS) is the most abundant and widely distributed glycosaminoglycan (GAG) in the human body. As a component of proteoglycans (PGs) it has numerous roles in matrix stabilization and cellular regulation. This chapter highlights the roles of CS and CS-PGs in the central and peripheral nervous systems (CNS/PNS). CS has specific cell regulatory roles that control tissue function and homeostasis. The CNS/PNS contains a diverse range of CS-PGs which direct the development of embryonic neural axonal networks, and the responses of neural cell populations in mature tissues to traumatic injury. Following brain trauma and spinal cord injury, a stabilizing CS-PG-rich scar tissue is laid down at the defect site to protect neural tissues, which are amongst the softest tissues of the human body. Unfortunately, the CS concentrated in gliotic scars also inhibits neural outgrowth and functional recovery. CS has well known inhibitory properties over neural behavior, and animal models of CNS/PNS injury have demonstrated that selective degradation of CS using chondroitinase improves neuronal functional recovery. CS-PGs are present diffusely in the CNS but also form denser regions of extracellular matrix termed perineuronal nets which surround neurons. Hyaluronan is immobilized in hyalectan CS-PG aggregates in these perineural structures, which provide neural protection, synapse, and neural plasticity, and have roles in memory and cognitive learning. Despite the generally inhibitory cues delivered by CS-A and CS-C, some CS-PGs containing highly charged CS disaccharides (CS-D, CS-E) or dermatan sulfate (DS) disaccharides that promote neural outgrowth and functional recovery. CS/DS thus has varied cell regulatory properties and structural ECM supportive roles in the CNS/PNS depending on the glycoform present and its location in tissue niches and specific cellular contexts. Studies on the fruit fly, *Drosophila melanogaster* and the nematode *Caenorhabditis elegans* have provided insightful information on neural interconnectivity and the role of the ECM and its PGs in neural development and in tissue morphogenesis in a whole organism environment.

## Introduction

This chapter highlights the roles of chondroitin sulfate (CS) and dermatan sulfate (DS)-proteoglycans (PGs) in neural biology, heparan sulfate (HS)-PGs were outside the scope of this review and thus are only briefly touched on. However many excellent reviews exist on HS-PGs and their interactions with extracellular matrix (ECM) components in neural development, neural function and potential in neural repair biology (Condomitti and de Wit, 2018; Zhang P. et al., 2018; Roppongi et al., 2020; Xiong et al., 2020; Kamimura and Maeda, 2021; Sakamoto et al., 2021). Roles for HS-PGs in model developmental organisms such as *Drosophila melanogaster* and *Caenorhabditis elegans* have also been reviewed (Díaz-Balzac et al., 2014; Blanchette et al., 2017; Kamimura and Maeda, 2017; Saied-Santiago et al., 2017) and the interested reader is referred to these excellent publications for further information.

With the identification of the multiple molecular determinants that provide neuronal connectivity, and with new insights into the modulatory extracellular information regulating axon guidance, neural network and synapse formation, a better understanding of the complexity that neurons face in a living organism is beginning to emerge. Attention is now returning to an ancient regulator of cell-cell interaction: the ECM (Dityatev and Schachner, 2006; Dityatev et al., 2010; Miyata and Kitagawa, 2017; Nicholson and Hrabětová, 2017; Ferrer-Ferrer and Dityatev, 2018; Quraishe et al., 2018; Cope and Gould, 2019; Long and Huttner, 2019; Chelyshev et al., 2020; Jain et al., 2020; Wilson et al., 2020; Carulli and Verhaagen, 2021; Kamimura and Maeda, 2021; Shabani et al., 2021; Su et al., 2021). Among the many matrix components that influence neuronal connectivity, recent studies on the CS-PGs and HS-PGs indicate these ancient molecules form dynamic scaffolds that not only provide a protective environment around cells but are also a source of directive cues that modulate neuronal behavior and synaptic plasticity in tissue development (Haylock-Jacobs et al., 2011; Hayes and Melrose, 2018; Hayes et al., 2018; Karamanos et al., 2018; Hayes and Melrose, 2020a; Shabani et al., 2021).

### Roles of GAGs in the Evolution of Life and Electrochemical Properties of Tissues

Glycosaminoglycans (GAGs) and PGs are ancient molecules that evolved over a 500 million year period of invertebrate and vertebrate evolution (Yamada et al., 2011). Natural selection processes ‘chose’ GAGs with molecular recognition, information storage and transfer properties. The PGs that populated the glycocalyx surrounding cells thus had cell instructive properties through their GAG side chains that interacted with morphogens, growth factors, cytokines, cell receptors, cell adhesion molecules and neurotrophic peptides facilitating regulatory roles in embryonic neural development. GAGs also have electro-chemical properties through their sulfate and carboxyl groups that are ionized at physiological pH. GAG-electroconductive gels in the sensory pores of the skin of elasmobranch fish species (i.e., sharks, rays, and skates) have the capacity to detect protons produced by the muscular activity of prey fish species and this equips them with the ability to undergo electro-location to detect prey species, even under highly turbid water conditions where these are not visible (Bellono et al., 2017). Such gels have ultrasensitive proton detection capability, this information is transferred to a sensory nerve interface in the skin pores and then to the brain stem for signal interpretation.

All GAGs have proton detection capability (Josberger et al., 2016; Selberg et al., 2019) and are ancient molecules that were present during the early stages of the evolution of life (Yamada et al., 2011). It has been proposed that proton electrochemical ion gradients across membranes drove cellular metabolism and energy production during early evolution (Lane, 2017). In prokaryotic evolution, GAGs were mainly unsulfated or poorly sulfated; however, when eukaryotic cells evolved, sulfated GAGs predominated. Evolution of membrane polarization became possible in eukaryotic cells and membrane energetics emerged (Wilson and Lin, 1980; Niven and Laughlin, 2008; Dibrova et al., 2012; Lynch and Marinov, 2017). Membrane polarization involves the controlled movement of ions across cell membranes, GAGs had fundamental roles to play in these processes through their proton binding properties. All cells in multicellular organisms utilize membrane polarization when undergoing cell signaling, adhesion, proliferation, migration, and cytokinesis. Some cells such as neurons have developed electrical processes to a high level of precision, and this is the basis of the generation of electrical impulses in neural networks that remotely control cells and tissues in higher animals. Further eukaryotic evolution resulted in the development of a glycocalyx around cells. This contained PGs containing GAG side chains with the ability to instruct cellular behavior. The development of pericellular and extracellular matrices populated by PGs facilitated the development of tissues with variable biophysical properties due to these PGs and their co-operative interactions with structural proteins thus driving specialization with the ECM. Neural networks subsequently evolved to control these tissues of increasing complexity. Neurons are highly energetic cell types that utilize Na(+)/K(+)-ATPase pumps to generate energy. This process also generates chemical and electrical gradients across cell membranes. This membrane polarization process is essential for cell signaling and is aberrantly controlled in neurological diseases. Examination of the ECM PGs that control these neural processes has uncovered valuable therapeutic targets (Soleman et al., 2013; Maćkowiak et al., 2014; De Luca and Papa, 2016; Miyata and Kitagawa, 2016; Yang, 2020; Dityatev et al., 2021).

Glycosaminoglycan were fundamental entities in the formation and regulation of neural networks and tissues and the control of cell behavior during morphogenesis, tissue development and in ECM remodeling in tissue repair (Melrose, 2019b). The sulfation patterns of GAGs have roles in cellular molecular recognition and the regulation of physiological processes (Melrose, 2019b). GAG sulfotransferases and glycosyl transferases in progenitor/stem cell niches support the assembly of GAGs of diverse structure and sulfation patterns and are important in the development of pluripotent stem cell lineages with migratory properties (Stanley, 2016). This allows these cells to participate in tissue development and tissue repair. Sulfate groups are important functional determinants on GAGs. Knock-out sulfotransferase and glycosyl transferase mice have demonstrated the important functional roles of GAGs in tissues (Stanley, 2016). Variable sulfation positions and densities on GAGs convey a range of functional attributes to tissues including an ability to act as electrical conduits to the cell. Sulfate groups are relatively bulky space-filling entities on GAGs, it was pertinent that all spatial orientations and permutations were explored during natural evolutionary selection processes to select GAGs with optimal interactivity. Natural selection forces thus explored many permutations of GAG structural form to optimize cell regulatory capability. Sulfate groups convey interactive molecular recognition and information transfer capability to GAGs and their interactions with growth factors, receptors, morphogens, ECM components, proteases, and protease inhibitory proteins regulate cell signaling processes in tissue morphogenesis and skeletogenesis. Knockout of glycosyl transferases, that are required for GAG assembly, has produced GAG-deficient mice (Stanley, 2016) that have allowed examination of the roles GAGs play in tissue form and function and how these regulate physiological processes in health and disease. The inherent charge transfer and storage properties of GAGs is a “glyco-code” that provides sophisticated cell instructive information (Gabius, 2018; Hayes and Melrose, 2018; Hayes et al., 2018; Kaltner et al., 2019).

#### CNS/PNS ECM PGs/GAGs, Cellular Regulation, and Neural Tissue Development

As already discussed, GAGs have electrochemical properties equipping them with cell regulatory abilities (Lane, 2017; Selberg et al., 2019). At the individual cell level, voltage gradients occur across cell membranes as so-called, action potentials (Strbak et al., 2016) which form part of the cell signaling and communication machinery of cells, i.e., membrane polarization-depolarization underlying the generation of electrical signaling in neural networks. Proton conductivity is important in many natural cellular processes including oxidative phosphorylation in mitochondria and energy production, uncoupling of membrane potentials during membrane polarization-de-polarization and neural potentiation, as well as in the priming of cells for proliferative events, apoptosis or cell migration (Wilson and Lin, 1980; Vellai et al., 1998; Niven and Laughlin, 2008; Dibrova et al., 2012; Lynch and Marinov, 2017). Electrochemical reactions control cell and tissue polarity and regulate cell behavior, ECM PGs facilitate electrocommunication between cells and their extracellular microenvironments. Cells sense changes in their microenvironments through micromechanical and electrochemical cues from the ECM allowing the cell to maintain a homeostatic tissue compositional balance thus providing optimal tissue functional properties (Guilak et al., 2021; Melrose et al., 2021). GAGs can detect proton gradients and are electroconductive entities that participate in microelectronic events during membrane polarization forming the basis of cell signaling (Wilson and Lin, 1980; Vellai et al., 1998; Niven and Laughlin, 2008; Dibrova et al., 2012; Lynch and Marinov, 2017). Neurons are particularly sensitive to electrostimulation in microelectronic events leading to polarization of the activated neuron cell membrane, however, membrane polarization occurs in all cells to some extent and is the basis of cell signaling during cellular attachment, migration and transmission of signals from cell to cell not only during development but also in neural repair and functional nerve recovery from trauma (Hortobágyi and Maffiuletti, 2011; Hayes and Melrose, 2020b). The GAG components of PGs participate in neurotrophic regulation of cellular movement in the development of neural networks and also in neural repair processes. A diverse collection of neuroregulatory molecules participate in these processes guided by cues from ECM PGs, which are discussed later in this chapter.

## The Chondroitin Sulfate and Dermatan Sulfate Components of Neural Proteoglycans

**FIGURE 1:**
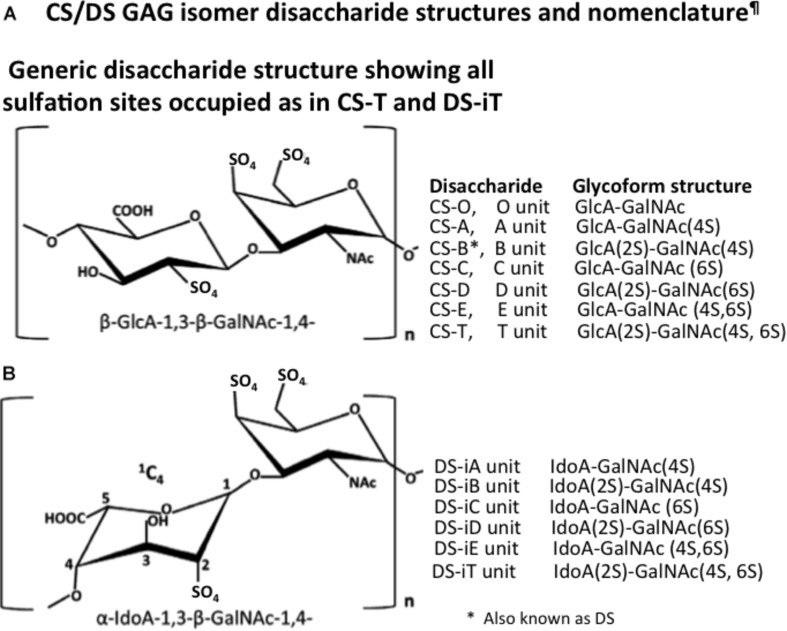
Structure of the CS/DS disaccharides **(A)** and nomenclature of the CS and DS disaccharide glycoforms **(B)** as proposed by Malavaki et al. (2008).

**FIGURE 2:**
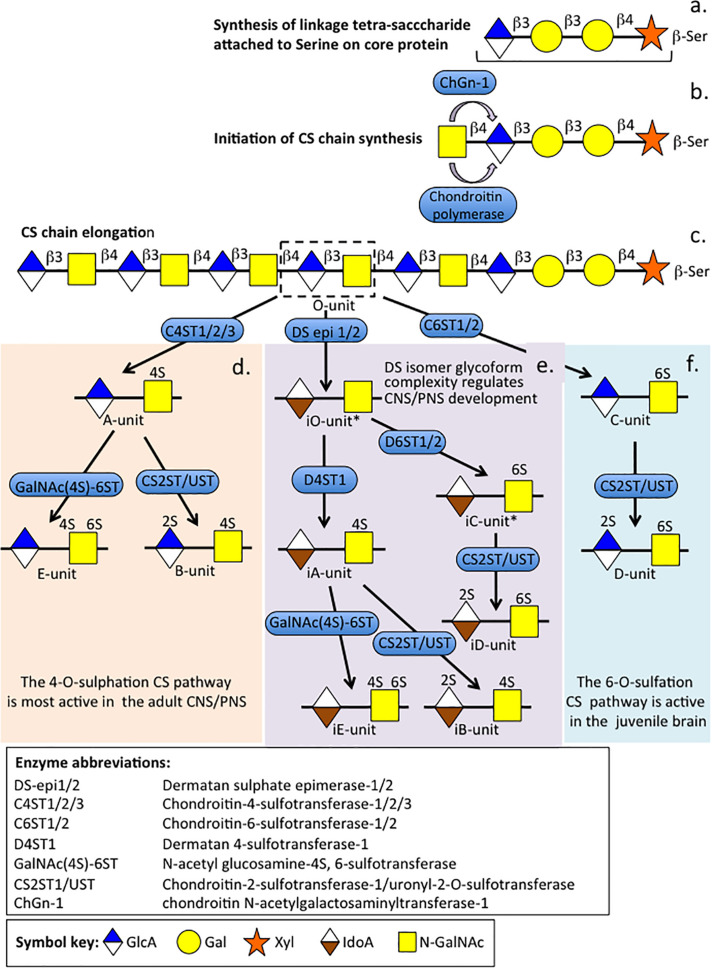
The biosynthesis of CS and DS chains showing their diverse disaccharide glycoforms that are functional in the juvenile and adult CNS/PNS and during the development and repair of the CNS/PNS. Biosynthesis of the tetrasaccharide linker sequence by addition of a xylose residue to a serine residue in the proteoglycan core protein followed by addition of two Galactose and a GlcA residue **(a)**. Initiation of CS chain elongation occurs by addition of a GalNAc residue by chondroitin *N*-acetylgalactosaminyltransferase-1 or chondroitinpolymerase **(b)**. Elongation of the CS chain occurs by sequential additions of GlcA and GalNAc to the nascent non-reducing terminus by chondroitin polymerases **(c)**. The CS chain is sulfated by chondroitin-4 and 6-sulphotransferases, or the GlcA residue of the *O*-disaccharide unit is epimerized to IdoA with inversion in structure from a β-D conformation to an α-L conformation followed by a series of sulphotransferases and uronyl-2-sulphotransferase to form the CS-A, CS-B, CS-C, CS-D, CS-E and DS-iA, DS iB, CS-iC, DS-iD, and DS-iE isoforms as shown **(d–f)**. The 4-*O*-sulfation pathway is most active in the adult brain **(d)** while various DS isoforms regulate brain development and repair processes **(e)**. The 6-sulfation pathway **(f)** is most active in the juvenile brain. The DS-iO and DS-iC units have yet to be confirmed. Figure modified from Malmström et al. (2012) and Miyata and Kitagawa (2017).

Chondroitin sulfate is the most abundant GAG of the human body and CS side chains are found on a diverse range of PGs. CS is a linear GAG consisting of *D*-glucuronic acid glycosidically linked to *N*-acetyl galactosamine to form repeat disaccharides assembled into CS side chains (Zhang, 2010) on PGs up to ∼20 kDa in size (Figure 1). D-glucuronic acid also undergoes epimerization and inversion in structure to form *L*-iduronic acid in the related GAG, DS also known as CS-B (Figure 2).

**FIGURE 3:**
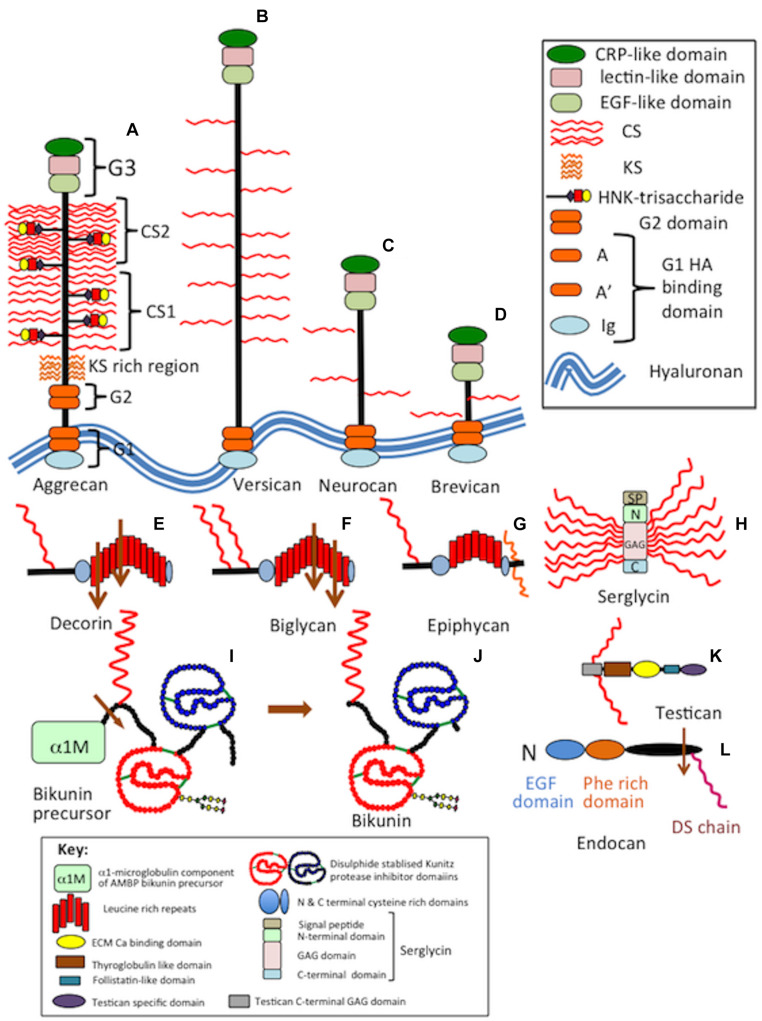
Composite schematic depicting the structural organization of secreted CS-proteoglycans in the CNS/PNS. The lectican proteoglycans aggrecan, versican, neurocan, brevican **(A–D)**, selected members of the small leucine repeat proteoglycans, decorin, biglycan and epiphycan **(E–G)**, serglycin **(H)**, bikunin precursor protein and bikunin **(I,J)**, testican **(K)** and endocan **(L)**.

**FIGURE 4:**
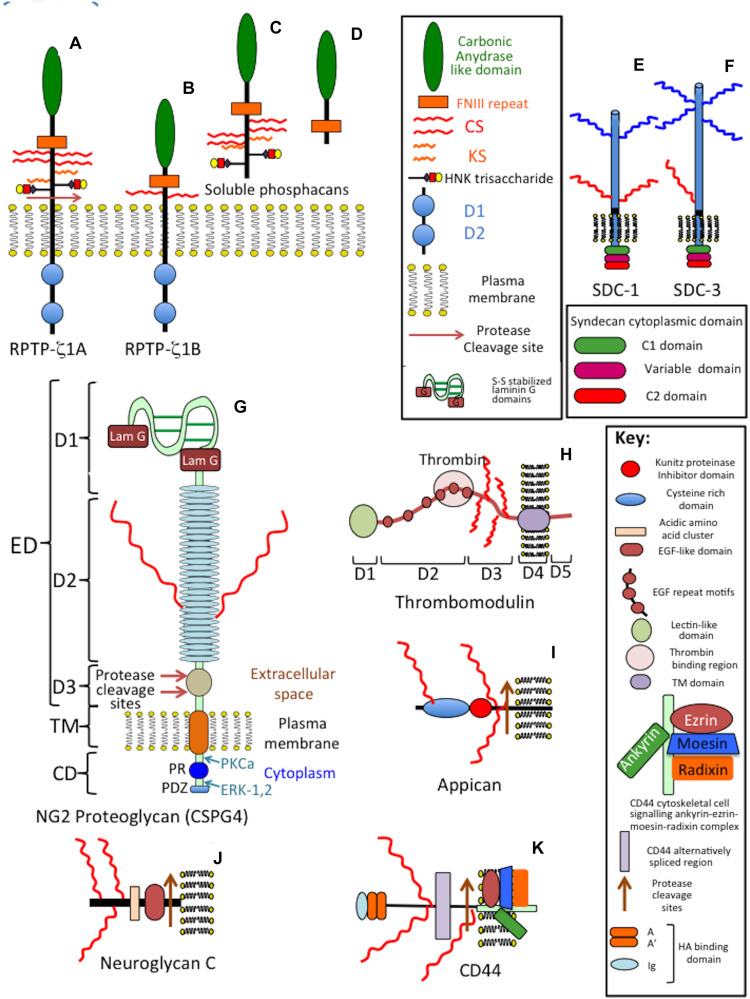
Composite schematic depicting the structural organization of cell membrane attached CS-proteoglycans in the CNS/PNS. Phosphacan precursor, receptor protein tyrosine phosphatase (RPTP)-1A and 1B forms **(A,B)** and soluble phosphacan released by proteolysis of **(A)** as shown **(C)**. A non-glycanated truncated phosphacan variant has also been described **(D)**. Syndecan-1 (SDC-1) **(E)** and syndecan-3 (SDC-3) **(F)**. The transmembrane form of NG2 proteoglycan (CSPG4) **(G)** and its soluble form released from cells by protease cleavage close to the cell membrane **(H)**. The soluble form of CSPG4 becomes lodged in the ECM through interactions mediated by its LamG N-terminal motifs and by interactions of its central cysteine-rich domain with type IV and VI collagen. Thrombomodulin showing its terminal lectin-like domain, six EGF repeat modules, thrombin binding region which allows it to act as an anti-coagulant in the brain microvasculature and transmembrane cell attachment domain **(I)**. Appican **(J)**, neuroglycan-C **(K)**, and the HA-receptor CD-44 **(L)** with protease cleavage sites that results in the generation of soluble forms of these proteoglycans. The CS-E chains of appican bind strongly to the growth factors midkine and pleiotrophin, neuroglycan-C binds to ephrin cell surface receptors resulting in induction of cell signaling mediated by the ephrin cytoplasmic regions while sCD44 binds to HA in the ECM through its disulphide stabilized A, A′ and Ig folds. Several CAMs also act as CS-PG receptors through interaction with the CS side chains of these PGs (see Figure 7).

**FIGURE 5:**
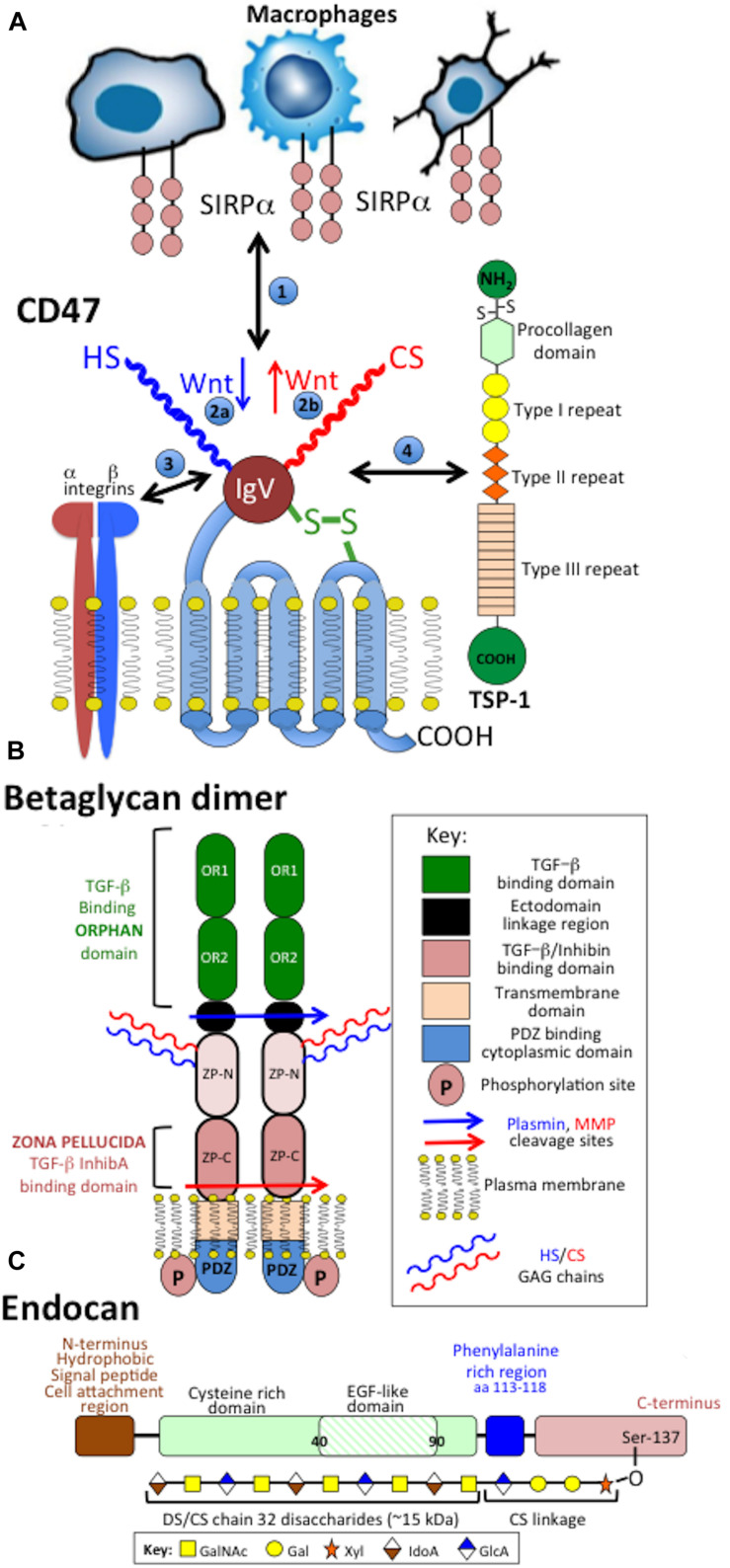
Schematic depictions of the structural organization of the transmembrane CS-PG receptors CD47 showing its interactions with macrophages, integrins and thrombospondin-1 (TSP-1) **(A)**, betaglycan dimer **(B)** and endocan **(C)**. Betaglycan forms an active dimer on the cell surface. Protease cleavage sites are indicated which release fragments of the betaglycan ecto-domain. Endocan has an EGF-like and a phenylalanine rich domain and a single CS/DS GAG chain attached to its C-terminus.

Chondroitin sulfate is *O*-sulfated at C-4 or C-6 of the GalNAc, whereas in DS GalNAc is almost exclusively 4-*O*-sulfated and minor proportions of L-idoA may be *O*-sulfated at C-2 (Malmström et al., 2012). The conversion of GlcA into IdoA is variable ranging from one to almost 99% conversion of GlcA to IdoA (Malmström et al., 2012). IdoA residues are not regularly distributed along the CS/DS chain and occur in blocks of ≥6 IdoA residues, alternating IdoA/GlcA units, or as isolated IdoA units interspersed within stretches of unmodified GlcA (Tykesson et al., 2018). DS epimerase-1 and dermatan 4-*O*-sulfotransferase-1 form complexes that generate long epimerized 4-*O*-sulfated blocks. The presence of idoA in CS/DS alters its properties since a more flexible chain is generated that is more able to explore spatial orientations that maximize binding opportunities with prospective ligands (Ferro et al., 1990). IdoA substituted CS/DS influences cellular properties, such as migration, proliferation, differentiation, angiogenesis and regulates cytokine/growth factor activities (Thelin et al., 2013). CS and DS occur in significant amounts in the brain and have important roles to play in CNS development. DS sulfate epimerase 2 (DS-epi2) is ubiquitously expressed in the infant brain whereas DS epimerase 1 (DS-epi1) expression is faint at all developmental stages (Akatsu et al., 2011). DS-epi2 but not DS-epi1 plays dominant roles in the epimerization of CS/DS and has crucial roles to play in postnatal brain development. CS/DS hybrid chains have roles in the development of the cerebellum with the expression of crucial disulfated CS/DS disaccharides spatiotemporally regulated by specific sulfotransferase enzymes (Mitsunaga et al., 2006). Ubiquitous expression of chondroitin 4-*O*-sulfotransferase-1 (C4ST-1) and C4ST-2 in the postnatal mouse brain contrasts with dermatan 4-*O*-sulfotransferase-1 (D4ST-1) and uronyl 2-sulfotransferase (UST) expression which are restricted to the developing cerebellum. The proportions of DS-specific, 4-sulfated IdoA-GalNAc (iA) and 2-sulfated IdoA-GalNAc (iB) produced by sequential D4ST-1 and UST activity has been shown to be highest in CS/DS chains isolated from the developing cerebellum with a 10-fold increase in iB evident. GlcA/IdoA(2-*O*-sulfate)-GalNAc(6-*O*-sulfate) (D/iD) and GlcA/IdoA-GalNAc (4,6-*O*-disulfate) (E/iE) levels, however, decrease to 50 and 30%, respectively, in the developing cerebellum. Thus IdoA-containing iA and iB and D/iD and E/iE units in CS/DS hybrid chains both have important roles to play in the development of the cerebellum and postnatal brain development. The diverse structures that are possible with CS provide multifunctional properties to CS-PGs (Abbott and Nigussie, 2020), with dynamic changes in CS structure providing adaptable regulatory properties to PGs in tissue development and in pathological conditions (Galtrey and Fawcett, 2007). CS-PGs as components of perineuronal nets (PNNs) have neuroprotective properties and regulate neural plasticity and cognitive learning through specific CS mediated interactions (Dyck and Karimi-Abdolrezaee, 2015). PGs are ubiquitous secreted ECM components (Figure 3) that also occur attached to cell surfaces either as transmembrane or glycophosphatidylinositol (GPI)-anchored structures, and intracellularly as granular deposits in some cells (Figures 4, 5). Perlecan is referred to as a HS-PG, however, in many tissues it is a hybrid CS/HS PG and is thus included in this review, particularly in view of its many interesting properties in neural tissues. The chain length of CS, 3D spatial presentation and density of its sulfate groups control its physicochemical and cell interactive and biological properties in tissues through interactions with a diverse range of ligands that regulate many physiological processes (Galtrey and Fawcett, 2007; Dyck and Karimi-Abdolrezaee, 2015). GAGs represent major ECM components of the brain, constituting up to 60% of its mass during early embryonic development and 20% in the adult central nervous system/peripheral nervous system (CNS/PNS). CS substituted PGs are one of the most abundant components of the CNS/PNS. HA is also a major component. HA has a simple structure and is the only non-sulfated GAG, but nevertheless has important biophysical properties which are important in the hydration and compartmentalization of the CNS/PNS. High molecular weight HA is anti-inflammatory, minimizes neuroinflammation and exhibits cell interactive properties that regulate cellular migration, proliferation and differentiation (Sherman et al., 2015). HA is also a component of the sub-ventricular and sub-granular dentate gyrus of the hippocampus which are two regions of the brain containing neuroprogenitor stem cell populations in niches known as fractones (Mercier et al., 2012; Mercier, 2016; Sato et al., 2019).

## Developmental Animal Models Used to Examine Neural Development and Regulation in a Whole Organism Environment

Studies on the fruit fly, *D. melanogaster* (FitzGerald et al., 2006; Nishihara, 2010; Losada-Perez et al., 2016; Davis et al., 2019) and the nematode *C. elegans* (White et al., 1976; Mulcahy et al., 2018; Schafer, 2018; Kovács et al., 2020; White, 2020) have provided insightful information on the role of the ECM and some of its specific components in neural development and tissue morphogenesis in a whole organism environment. White et al. (1986) undertook the first complete mapping study of the nematode’s nervous system using manual reconstruction of serial electron micrographs, to characterize the morphology of each of the 302 neurons in the adult nematode and their interconnected chemical and electrical synapses. This study is an invaluable guide on the *C. elegans* neural network and has significantly influenced studies on behavioral neurobiology and network science. The nervous system of *C. elegans* has a total of 302 invariantly organized neurons, that have been grouped into 118 categories. Neurons in *C. elegans* have simple morphologies displaying few, if any, branches and are generally highly connected through local synaptic connections with neighboring neurons, ∼ 5000 chemical synapses, 2000 neuromuscular junctions and 600 gap junctions have been identified in *C. elegans*. The specific patterns and functional properties of electrical synapses of the *C. elegans* nervous system have been systematically examined through a genome- and nervous-system-wide analysis of the expression patterns of the invertebrate electrical synapse constituents, the innexins (Hu, 2007; Güiza et al., 2018; Bhattacharya et al., 2019). Innexins are transmembrane proteins that form gap junctions in invertebrates. Highly complex expression patterns are evident throughout the nervous system, and when animals enter an insulin-dependent arrest stage due to exposure to harsh environmental conditions, termed the dauer stage, *C. elegans* larvae undergo Dauer arrest at the second molt (Cassada and Russell, 1975). Many insights into the signaling pathways and the molecular mechanisms that govern this developmental transition have been uncovered in the last decade. Dauer pheromone the major physiologic signal that promotes Dauer arrest, has been purified, identified, and synthesized and the vast majority of Dauer regulatory genes are now known (Jeong et al., 2005). Dauer pheromone (daumone, ascr#1) is the first *C. elegans* pheromone identified. Electrical synapse remodeling is responsible for the altered locomotory and chemosensory behavior of Dauer affected neurons (Starich et al., 2009). These neurons are regulated by a mechanism that involves FoxO transcription factors that mediate dynamic innexin expression plasticity in a neuron-type- and environment-specific manner (Hobert, 2010; Amran et al., 2021). *C. elegans* is a useful model for the examination of developmental processes regulated by ECM components (Hobert, 2010; Amran et al., 2021). HSPGs and chondroitin proteoglycans (CPGs) both have prominent roles to play in developmental processes in *C. elegans* (Saied-Santiago et al., 2017). Most ECM molecules in *C. elegans* are conserved and are homologues to mammalian proteins, however, fewer ECM protein isoforms are present and these exhibit less redundancy compared to in mammalian tissues. Furthermore, mutations in *C. elegans* produce comparable developmental defects to those evident in mammals. The small size, short lifespan of 3–4 days and defined neural interconnectivity of the *C. elegans* nervous system facilitate its use in systematic functional studies that have yielded valuable insights into neuronal differentiation (Chisholm and Jin, 2005). This information is also relevant to more complex neural systems since the basic cellular machinery of synaptic transmission is highly conserved across species. In *C. elegans*, 719 out of ∼20,000 genes (∼4%) of its genome encode matrisome proteins, including 181 collagens, 35 glycoproteins, 10 proteoglycans, and 493 matrisome-associated proteins, 173 out of the 181 collagen genes are unique to nematodes and are predicted to encode cuticular collagens (Teuscher et al., 2019).

Comparative genomic studies on human and *C. elegans* has identified orthologous genes in *C. elegans* that have comparable regulatory properties to human genes and demonstrated mechanisms relevant to human biology. *C. elegans* represents an experimental system that can be used in genetic approaches to address biological questions relevant to human development, physiology, and disease. Genetic based phenotype screens have identified *C. elegans* genes homologous to human disease-associated genes, and fundamental properties about their roles and mechanisms of action in pathological human tissues. Reverse genetic methods developed in *C. elegans* have expanded the repertoire of genetic approaches to examine the roles of specific genes in human disease processes. These methods have the ability to phenocopy loss-of-function mutations by feeding worms bacteria expressing double-stranded RNA, RNAi. Studies in *C. elegans* have also facilitated genome-wide screens or screens specifically targeting human disease genes and the large-scale generation of deletion or point mutations for functional genetic studies (Kim et al., 2018).

The *Drosophila* matrisome consists of 641 genes, 27 are homologs/orthologs to human core matrisome genes, 219 are homologs/orthologs to mammalian matrisome genes and the remaining 395 are genes are specific to *Drosophila* (Davis et al., 2019). Comparative genomic analyses have been undertaken between human and *Drosophila* genomes and core promoter regions. Although fruit flies have a genome that is 25 times smaller than the human genome, many fruit fly genes have comparable genes in humans that control the same biological functions. Twelve fruit fly genome sequences are available in the FlyBase database^1^, a collaboration of Harvard, Cambridge, Mass.; Indiana, Bloomington; and the University of Cambridge, United Kingdom. The fruit fly genome sequences are also available from NIH’s National Center for Biotechnology Information^2^, European Molecular Biology laboratory’s Nucleotide Sequence Database, EMBL-Bank^3^, and DNA Data Bank of Japan, DDBJ^4^ (FitzGerald et al., 2006).

As found in higher organisms, PGs have important roles in cellular regulation critical to the development of metazoan organisms. *C. elegans*, however, produces a large amount of non-sulfated chondroitin (Chn) in addition to a small amount of low sulfation CS. Until recently, *C. elegans* was known to express nine Chn/CS-PGs dissimilar to vertebrate CS-PGs. A recent glycoproteomic study identified 15 additional CPGs in *C. elegans* and three of these were homologous to human proteins, thus selected CS-PG core proteins appear conserved throughout evolution (Noborn and Larson, 2021). Bioinformatic analysis of primary amino acid sequence data identified a broad range of functional domains in these *C. elegans* PGs, thus specific PG core protein mediated functional properties appeared to have evolved early in metazoan evolution.

*Drosophila melanogaster* has also proved to be a useful model for the investigation of developmental GAG functions *in vivo* confirming *in vitro* findings. HS and CS GAG side chains of PGs are structurally conserved between *Drosophila* and mammals, including humans. Mutant and RNAi fruit flies show that HS-PGs and CS-PGs play key roles in the regulation of developmental signaling pathways involving FGF, Wingless (Wg)/Wnt, Hedgehog, and Decapentaplegic (Dpp, a ligand of the TGFβ superfamily). Glycosyl transferases, sulfotransferases, sugar-nucleotide transporters including 3′-phosphoadenosine 5′-phosphosulfate (PAPS) transporters, all have important roles to play in GAG biosynthesis and the functional status of PGs in neuronal development and maintenance (Nishihara, 2010). It should be noted, however, that the major non-sulfated Chn in *C. elegans* has crucial roles in embryonic cell and tissue development and tissue morphogenesis. Since Chn is present on mammalian PG core proteins along with CS no studies have been possible to specifically target the functional roles of Chn in these PGs, however, insights into the biological properties of Chn in mammals can be gleaned from the use of Chn as a drug in the treatment of osteoarthritis (OA) (Singh et al., 2015). A meta-analysis of 43 reviews which analyzed the use of Chn for the treatment of OA and alleviation of joint pain yielded moderate to inconclusive results. However, Chn elicited a significant improvement in the anti-inflammatory profile of synoviocytes and chondrocytes in an OA model analyzed by multiplex and Western blot analysis. Chn significantly decreased the levels of several pro-inflammatory cytokines (IL-1β, IL-5, 6, 7, 9, 15, 17), anti-inflammatory cytokines (IL-4, IL-10), chemokines (IL-8, MCAF, MIP-1a, MIP-1b, RANTES) in synovial fluid samples and decreased expression of the OA biomarkers MyD88 and MMP-13 (Vassallo et al., 2021). Evidence therefore exists that Chn displays anti-inflammatory properties in mammalian tissues. However in *C. elegans* where Chn predominates over CS the biological properties of Chn have more profound effects on neural biology. A number of studies have demonstrated the fundamental biological roles played by Chn, CS, and DS, attached to the core proteins of cell surface and ECM PGs (Sugahara et al., 2003). PGs decorated with CS, DS, or HS have diverse roles in growth factor, morphogen and cytokine-mediated cell signaling through cellular receptors that play critical roles in the development of the CNS (Melrose et al., 2021). As discussed later in this chapter, these functions of PGs are closely associated with GAG sulfation patterns. Surprisingly, non-sulfated Chn is indispensable in the morphogenesis and cell division of *C. elegans*, as revealed by RNA interference experiments of the recently cloned *chondroitin synthase* gene and by the analysis of *squashed vulva* (sqv) gene mutants. It should be noted that while orthologous forms of human perlecan/HS-PG2 exist in *C. elegans* (UNC-52) and *D. melanogaster* (Trol) these have a different modular structure to human perlecan and are devoid of domain-I and thus they lack HS substitution (Celestrin et al., 2018). Thus the interactive properties provided by the HS chains of human perlecan do not occur in these orthologs and the interactive properties conveyed by these form of perlecan is due to modular components in their core proteins (Condomitti and de Wit, 2018; Martinez et al., 2018).

### The Instructional Properties of CS- and HS-PGs in Neural Development and Tissue Morphogenesis in *Caenorhabditis elegans*

Blocking of chondroitin synthesis results in cell proliferative defects in early embryogenesis in *C. elegans* and leads to early embryonic death (Mizuguchi et al., 2003). Mutations in eight sqv genes in *C. elegans* causes defects in embryonic cytokinesis and in vulval morphogenesis during postembryonic development. Sqv-1, 2, 3, 4, 6, 7, 8 control CS and HS biosynthesis, while sqv-5 encodes a bifunctional glycosyltransferase responsible for the biosynthesis of Chn, but not HS (Hwang et al., 2003a, b). Sqv mutations in *C. elegans* have lethal consequences due to disruption in cell proliferation and the lack of the formation of an extracellular space between the egg and the eggshell, apparently due to disruption in the normal GAG containing ECM structures. Cloning and characterization of the sqv-2 and -6 genes showed that sqv-6 encoded a protein similar to human xylosyltransferase, while sqv-2 encoded a protein similar to human galactosyltransferase II. SQV-6 and SQV-2 proteins act in concert with other SQV proteins to catalyze the stepwise formation of the PG core protein linkage tetrasaccharide GlcAβ1,3Galβ1, 3Galβ1,4Xylβ-*O*-(Ser), common to CS and HS (Hwang et al., 2003a, b). This linkage tetrasaccharide acts as an acceptor molecule for the assembly of the CS and HS chains. Chain elongation is initiated by the addition of GlcNAc or GalNAc, with the former addition resulting in the biosynthesis of HS chains by sequential additions of GlcA and GlcNAc, while if GalNAc is the initial sugar added to the acceptor group this results in the synthesis of CS chains. These GAG chains are sulphated in a later biosynthetic stage at various positions on GlcNAc or GalNAc by specific sulfotransferases. GlcA can also be epimerised to IdoA and sulphated at O-2 in HS chains. HS chains can be sulphated at multiple positions. CS sulfation is an important functional determinant in the regulation mammalian neural tissue development and repair, however, in *C. elegans* and *D. melanogaster* the Chn chains are not sulphated but nevertheless have essential roles to play in early embryonic development and tissue morphogenesis in later developmental stages.

Vertebrates produce multiple CS-PGs with important roles in development and the mechanical performance of tissues. The Chn chains in *C. elegans* are not sulfated, but nevertheless they still play essential roles in embryonic development and vulval morphogenesis (Olson et al., 2006). *C. elegans* Chn PG core proteins, do not share sequence similarities with PGs from *D. melanogaster* or *Hydra vulgaris*. The *C. elegans* CPG-1 and CPG-2 PGs are expressed during embryonic development and bind chitin, which may have a structural role to play in the egg (Olson et al., 2006). Chitin is a widespread polymer in nature and is a polymer composed of *N*-Acetyl glucosamine. Chitin is a primary component of fungal cell walls, the exoskeletons of arthropods such as crustaceans and insects and the scales of fish. Depletion of CPG-1/CPG-2 results in multinucleated single-cell embryos in *C. elegans*, this is also observed with depletion of the SQV-5 chondroitin synthase protein, Chn chains of CPG1/CPG2 play essential roles in cytokinesis. This is achieved through regulation of GAG biosynthetic enzymes. *C. elegans* microRNA mir-79, an ortholog of mammalian miR-9, controls sugar-chain homeostasis by targeting two proteins in the PG GAG biosynthetic pathway: a chondroitin synthase (SQV-5; squashed vulva-5) and a uridine 5′-diphosphate-sugar transporter (SQV-7). Loss of mir-79 causes neurodevelopmental defects through dysregulation of SQV-5 and SQV-7. This results in a partial shutdown of HS biosynthesis that effects the LON-2/glypican pathway and disrupts neuronal migration. MicroRNA thus represents a regulatory axis that maintains PG homeostasis. Sqv genes 1–8 control the invagination of vulval epithelial cells, normal oocyte formation and embryogenesis. Sequencing of sqv-3, sqv-7, and sqv-8 genes indicated potential roles for the proteins they encode in glycolipid or glycoprotein biosynthesis. sqv-3, -7, and -8 affect the biosynthesis of GAGs and the bioactivity of PGs establishing their essential roles in tissue morphogenesis and pattern development in *C. elegans* (Bulik et al., 2000).

Sulfation of PG GAG side chains has critical roles to play in the cell regulatory properties of PGs and their roles in many essential physiological processes. Sulfation reactions involves activated sulfate, and the universal sulfate donor 3′-phosphoadenosine 5′-phosphosulfate (PAPS). In animals, PAPS is synthesized from ATP and inorganic sulfate by PAPS synthase, genetic defects in PAPS synthase 2, one of two PAPS synthase isozymes, causes dwarfism. In order to better understand the developmental role of sulfation in tissue PGs, a *C. elegans* PAPS synthase-homologous gene, pps-1 has been cloned and the depleted expression of its product, PPS-1 examined (Dejima et al., 2006). PPS-1 protein exhibits specific roles in the formation of PAPS *in vitro*. Disruption of the pps-1 gene by RNAi methods causes widespread developmental tissue defects, a decrease in GAG sulfation in the pps-1 null mutant exhibits larval lethality. Sulfation is essential for normal growth and the integrity of the epidermis in *C. elegans* and has been used as a model to demonstrate the role of HS modifications in a defined biological process. Genetic analyses suggest that syndecan/sdn-1 and HS 6-*O*-sulfotransferase, hst-6, function in a signaling pathway and glypican/lon-2 and HS 2-*O*-sulfotransferase, hst-2, function in a parallel pathway. HS modifications may be part regulated at the level of tissue expression of genes encoding for HS-PGs and HS modifying enzymes. There is a delicate balance in such HS modifications that may deleteriously effect cell migration, HS is a critical regulator of cell signaling in normal development and disease. HS-PGs have roles in the structural organization of neurochemical synapses, involving interactions with the core protein as well as the HS side chains (Cizeron et al., 2021). Specific modifications to HS contribute to a sugar code which provides specificity to synaptic interactions. SDN-1 is a unique *C. elegans* syndecan ortholog found in synaptic junctions. 3-*O* sulfation of SDN-1 maintains the ECM protein punctin/MADD-4/ (MAP kinase-activating death domain protein) that defines the synaptic domains, however, in mammals 3-*O* sulfation is a rare modification in HS despite the seven HS modifying enzymes that can produce 3-*O* sulfation.

Punctin/MADD-4, a member of the ADAMTSL ECM protein family, is a synaptic organizer in *C. elegans.* MADD is an enzyme encoded by the MADD gene. The Ig-like domain of MADD is the primary determinant for N-MADD-4B interactions with NLG-1 *in vitro* (Platsaki et al., 2020). At GABAergic neuromuscular junctions, the short isoform MADD-4B binds the ectodomain of neuroligin (NLG-1), which is also a postsynaptic organizer of inhibitory synapses (Tu et al., 2015). Proteolysis of MADD-4B generates *N*-MADD-4B, which contains four thrombospondin domains and one Ig-like domain that bind NLG-1 (Maro et al., 2015). A second processing event eliminates the C-terminal Ig-like domain of N-MADD-4B and its ability to bind NLG-1. The death domain of the type 1 tumor necrosis factor receptor (TNFR1) mediates the downstream effects of TNF. MADD interacts with TNFR1 residues and is a component of the TNFR1 signaling complex. The MADD death domain stimulates ERK and c-JUN N-terminal kinase MAP kinases inducing phosphorylation of cytosolic phospholipase A2. Thus, MADD links TNFR1, MAP kinase activation and arachidonic acid release, which may explain the pleiotropic effects of TNF.

Growth cones facilitate the repair of damaged neural tissue by promoting axon regeneration, syndecan, is required for growth cone function during axon regeneration in *C. elegans* (Edwards and Hammarlund, 2014; Gopal et al., 2016, 2021). In the absence of syndecan, regenerating growth cones are unstable and they collapse, impeding regrowth to target cells. Syndecan has two distinct functions during axon regeneration: (i) axon guidance requiring its HS-dependent expression outside the nervous system (ii) intrinsic growth cone stabilization mediated by the SDC core protein independently of HS.

### The Instructional Properties of CS- and HS-Proteoglycans in Neural Development and Tissue Morphogenesis in *D. melanogaster*

Blocking Chn synthesis results in defects in cytokinesis and embryogenic development in *D. melanogaster* leading to early embryonic death. This demonstrates the essential developmental roles Chn plays in *Drosophila* embryonic cytokinesis and cell division. *Drosophila* has proved to be a useful model for the investigation of developmental GAG functions *in vivo* confirming *in vitro* findings with GAGs (FitzGerald et al., 2006; Nishihara, 2010). HS and CS GAG side chains of PGs are structurally conserved between *Drosophila* and mammals, including humans. CS sulfation is an important functional determinant in the regulation of mammalian neural tissue development and repair; however, in *C. elegans* and *D. melanogaster* the Chn chains are not sulfated but, nevertheless, have essential roles to play in early embryonic development and tissue morphogenesis in later developmental stages.

Windpipe (Wdp) is a novel CS-PG recently identified in *Drosophila*. Wdp is a single-pass transmembrane protein with leucine-rich repeat (LRR) motifs and has three extracellular CS chain attachment sites (Takemura et al., 2020). Wdp modulates the Hedgehog (Hh) cell signaling pathway. In the wing disk, overexpression of Wdp inhibits Hh signaling an effect that is dependent on its CS chains and protein interactive LRR motifs. The JAK/STAT (Janus Kinase/Signal Transducer and Activator of Transcription) signaling pathway regulates adult stem cell activities and is essential for the maintenance of intestinal homeostasis in *Drosophila*. Wdp interaction with the receptor Domeless (Dome) promotes its internalization and lysosomal degradation. Wdp thus acts as a negative feedback regulator of JAK/STAT cell signaling and is a novel regulatory component of JAK/STAT signaling in *Drosophila* adult intestines (Ren et al., 2015).

Glycosaminoglycans such as HS and CS have roles in intercellular signaling thus disruptions of genes encoding enzymes that mediate GAG biosynthesis have severe consequences in *Drosophila* and mice. Mutations in the *Drosophila* gene *sugarless*, encoding a uridine diphosphate (UDP)-glucose dehydrogenase, impairs developmental signaling through the Wnt family member Wingless, and FGF and Hedgehog signaling pathways. Undersulfated and oversulfated CS chains are implicated in neural development, cloning of a chondroitin synthase homolog in *Caenorhabditis elegans* and depletion in Chn expression results in defects in cytokinesis in early embryogenesis and early embryonic death demonstrating the essential role Chn plays in early developmental processes (Mizuguchi et al., 2003).

*Drosophila* has an NG2 homologue called kon-tiki (kon), that promotes CNS repair (Losada-Perez et al., 2016). Crush injury upregulates kon expression and induces glial cell proliferation and differentiation by activating glial genes and prospero (pros). Negative feedback loops with Notch and Pros allow Kon to drive the homeostatic regulation of neuronal repair with modulation of Kon levels in glia, potentially preventing or promoting CNS repair (Losada-Perez et al., 2016). The interplay between Kon, Notch, and Pros is therefore essential in neural repair in *Drosophila*. Prospero homeobox protein-1 is encoded by the *PROX1* gene in humans. This pan-neural protein has essential roles to play in the proper differentiation of neuronal lineages and in the expression of genes in the *Drosophila* CNS. Prospero is a sequence-specific DNA-binding protein that can act as a transcription factor through interaction with homeodomain proteins to differentially modulate their DNA-binding properties (Hassan et al., 1997; Yousef and Matthews, 2005). Functional interactions between Prospero and homeodomain proteins is supported by observations showing that Prospero, together with the homeodomain protein, Deformed, are required for proper regulation of a Deformed-dependent neural-specific transcriptional enhancer (Hassan et al., 1997). The DNA-binding and homeodomain protein-interactive properties of Prospero are localized in its highly conserved C-terminal region.

Syndecan (Sdc) is a conserved transmembrane HS-PG bearing CS chains on its ectodomain. In vertebrates, this extracellular domain of Sdc is shed and acts as a soluble effector of cellular communication events, and the Sdc cytoplasmic domain participates in intracellular signaling needed to maintain epithelial integrity. In *Drosophila*, Sdc has been shown to be necessary for Slit signaling-dependent axonal guidance during CNS development (Chanana et al., 2009). Sdc acts in a cell-autonomous manner in Slit-receiving cells and that its membrane-anchored extracellular domain is sufficient to mediate Slit signaling. The HS-PG Dally-like protein (Dlp), which lacks CS on its extracellular domain, can only partially substitute for Sdc function but its activity is not restricted to the Slit target cells. Sdc and Dlp act in a cooperative but non-redundant manner in neural tissues with Dlp transferring Slit from its site of expression to the target cells, where it interacts with CS-modified Sdc.

### *Caenorhabditis elegans* Netrins and Neural Development

UNCoordinated-6 (UNC-6) was the first *C. elegans* member of the netrin family that was discovered (Krahn et al., 2019). UNC-6 shares homology to human netrin-1, and is a key signaling molecule in the regulation of directional axonal migration in nematodes (Krahn et al., 2019). Similar to netrin-1, UNC-6 interacts with multiple receptors to guide axonal migration (Moore et al., 2007; Rajasekharan and Kennedy, 2009; Ogura et al., 2012). Netrin is a key guidance protein regulating the orientation of axonal growth during neural network formation in *C. elegans*. LON-2/glypican, modulates UNC-6/netrin signaling through interactions with the UNC-40/DCC (deleted in colorectal carcinoma) receptor (Yang et al., 2014). LON-2 expressed on the cell surface in the intestine and hypodermis in *C. elegans* and in *D. melanogaster* promotes growth factor binding in several developmental processes, negatively regulating the TGF-β receptor signaling pathway and BMP-like signaling that regulates tissue growth and body length. N-terminal sequencing of the signal peptide of LON-2, identified a 14 cysteine domain of functional importance, SGXG GAG attachment site and C-terminal GPI anchor site showing that LON-2 is a member of the glypican family (Eisenhaber et al., 2000; Gumienny et al., 2007). The other *C. elegans* glypican, *gpn-1*, has no significant effect on the body size (Blanchette et al., 2015). Null mutations in *C. elegans* genes encoding HS biosynthetic enzymes that process the HS side chains of LON-2, significantly reduce body size. *hse-5*, *hst-2*, and *hst-6* encode *C. elegans* counterparts to mammalian glucuronyl C5-epimerase, 2 *O*-sulfotransferase, and 6 *O*-sulfotransferase, respectively. This demonstrates the important functional role HS plays in neural development in *C. elegans* and the importance of the HS sulfation patterns for this activity (Bülow and Hobert, 2004; Gysi et al., 2013; Díaz-Balzac et al., 2014; Saied-Santiago et al., 2017). HS chains of HS-PGs carry multiple structural modifications due to sulfation and epimerization of GlcA that influence their ligand binding properties. This is why HS-PGs have such diverse effects in tissue and axonal development. The core proteins of *C. elegans* SDN-1 and glypican/LON-2 and HS modifying enzymes thus both have roles in axonal guidance through interactions with UNC-6/Netrin (Rhiner et al., 2005). *C. elegans* SDN-1/syndecan control of neural migration and axonal guidance also occurs through regulation of Slit/Robo signaling in parallel with C5-epimerase HSE-5, and/or the 2O-sulfotransferase HST-2 activity, which provide distinct regulatory HS modification patterns on SDN-1.

## Mammalian Neuronal Proteoglycans

Neural PGs occur as large and small extracellular, cell surface and intracellular components. The salient features of neural PGs are summarized in Table 1 and their structural organizations are shown schematically in Figures 3–5.

**TABLE 1:** Extracellular, cell associated and intracellular CS-proteoglycans of the CNS/PNS.

Protein	Distribution	Roles	References
**Large lectican neural proteoglycans**			
Aggrecan (ACAN) (CSPG1)	Present as diffuse ECM component between the dense ECM of PNNs which contain HA and the lecticans	Roles in tissue hydration, space-filling in CNS/PNS, neuroprotective in PNNs, synapse formation, roles in synaptic plasticity memory, cognitive learning	Kiani et al., 2002; Hayes and Melrose, 2020a
Versican (VCAN) (CSPG2)	Widespread in CNS/PNS occurs as V0, V1, V2, V3 isoforms	Promotes proliferation, differentiation, cell migration, tissue development, repair, tissue morphogenesis. G1 and G3 stimulate cell migration. Versikine G1 fragment of Versican V2 is an Alarmin in innate immunity with TLR4.	Schmalfeldt et al., 1998; Wu et al., 2004; Xiang et al., 2006; Schmitt, 2016; Islam and Watanabe, 2020
Neurocan (NCAM) (CSPG3)	150 kDa lectican CS-PG Widespread CNS/PNS PG	Interacts with HA, NCAM, modulates cell binding, regulates neurite outgrowth through interactions with Sdc-1, Gpc-3, and PTN.	Margolis and Margolis, 1994; Margolis et al., 1996; Rauch et al., 2001; Sullivan et al., 2018; Schmidt et al., 2020
Brevican (CSPG7)	Widespread CNS/PNS PG, present in the post synaptic gap where it may relay neurotransmitters to adjacent communicating neurons	Binds to astrocytes and neurons regulates axon and dendritic maturation, upregulated in glial scars. GPI anchored brevican described. BEHAB is a bioactive fragment that promotes glioblastoma development.	Yamaguchi, 1996; Gary and Hockfield, 2000; Matthews et al., 2000; Giamanco and Matthews, 2020
**Large non-lectican neural proteoglycans**			
RPTPR-ζ Phosphacan	Populations of phosphacan contain variable levels of CS, KS, or HNK-1 trisaccharide substitution Cell surface (RPTPR-ζ) and soluble PG (phosphacan) forms	RPTPR-ζ, single pass type 1 TM PG-phosphatase receptor, regulates SC repair and neurogenesis, soluble phosphacan ectodomain, has truncated forms with neurite outgrowth activity.	Garwood et al., 2003; Faissner et al., 2006; Eill et al., 2020
NG2 (CSPG4)	Widely distributed TM Oligodendrocyte PG, a soluble form is released from cell by proteases	Stimulates endothelial cell proliferation, sequesters FGF-2 and PDGF. Collagen VI receptor activates FAK/ERK1/ERK2 signaling. Up-regulated in SCI and tumors.	Jones et al., 2002; Wigley et al., 2007; Tamburini et al., 2019
Betaglycan	250–280 kDa CS/HS TM homodimeric PG	Binds inhibin, FGF-2, Wnt TGF-β HS inhibits and CS promotes Wnt signaling. Fragments of betaglycan are released by plasmin and MMPs. binding to inhibin antagonises activin signaling	Lewis et al., 2000; Gray et al., 2001; Bilandzic and Stenvers, 2011; Miller et al., 2012; Kim et al., 2019
Perlecan (HSPG2)	HS/CS hybrid PG of BBB, NMJ, BM, and of fractone stem cell niche	Stabilizes BBB and motor neuron endplate BM. Regulates neuroprogenitor proliferation by FGF-2 in SVZ and dentate gyrus fractones. Domain V promotes neurogenesis, BBB repair	Cho et al., 2012; Kerever et al., 2014; Celestrin et al., 2018
**Small neural proteoglycans**			
Neuroglycan-C (CSPG5, CALEB) Acidic, Leu-rich EGF Brain PG	Part-time TM PG, growth and differentiation factor involved in neuritogenesis	Core protein EGF domain, CS-E side chains, ligand for ErbB3. Binds PTN to promote neurite outgrowth.	Watanabe et al., 1995; Kinugasa et al., 2004; Shuo et al., 2007; Nakanishi et al., 2010
Syndecan-1. Syndecan-3 (Sdc1, Sdc3)	TM CS/HS CNS/PNS PGs	Sdc-1 and 3 interact with midkine, and PTN, roles in neural development, neurite outgrowth, neural proliferation	Couchman et al., 2015; Gopal et al., 2016
Decorin (DCN)	∼92.5 kDa class I SLRP containing one N-terminal CS or DS chain, 42 kDa core protein, and 12 LRRs. Widely distributed in CNS/PNS and around gliotic scars	Pluripotent, regulates IL-1, VEGF2, CTGF, TGF-β EGF, IGF-1, participates in ECM assembly, cell growth, differentiation, proliferation, adhesion, and migration. Regulates inflammation, fibrosis, fibrillogenesis, TGF-β bioavailability. “Mayday” and “Decorunt” DCN fragments. Mayday attracts MSCs into tissue defects	Lee et al., 2014; Zhang W. et al., 2018
Biglycan (BGN)	∼200 kDa class I SLRP containing two N-terminal CS or DS chains and a 42 kDa core protein and 12 LRRs	Structural ECM component, TLR-2, 4 interactive DAMP Alarmin protein in innate immunity, modulates growth factor (TGF-β, TNFα, BMP-2, 4, 6; WISP-1) and cytokine functions and is a stabilizing component of synapses. also interacts with complement system. An *en silico* generated BGN fragment (P2K) is a TGF-β inhibitor.	Amenta et al., 2012; Nastase et al., 2012; Chen et al., 2018, 2020; Xie et al., 2020
Epiphycan (EPN) DSPG3,PG-Lb	Epiphycan (EPN) also known as DSPG3 or PG-Lb is a CS/DS substituted 133 kDa SLRP with a 46 kDa core protein and contains 7 LRR repeats. EPN is a Cochlear SLRP	Epiphycan (EPN) is a CS/DS 133 kDa SLRP with a 46 kDa core protein and c7 LRR repeats. EPN has roles in auditory cochlear neuronal interactions, EPN deficiency leads to deafness.	Johnson et al., 1997; Hanada et al., 2017
Appican (APP)	APP is a 100–110 kDa type I TM PG alternatively spliced APLP2 is also found in neural tissues	APP has neuroregulatory properties through PTN: CS-E interactions	Pangalos et al., 1996
Bikunin/ITI	Bikunin is the light chain of ITI and has a mass of 25–26 kDa, contains a single CS chain.	Multifunctional Kunitz protease inhibitor PG, anti-metastatic, immune-modulator, growth promoter. Stabilizes HA by crosslinking ITI HCs to HA	Hamasuna et al., 2001; Lord et al., 2013, 2020
Serglycin (SGN)	Mast cells, platelets, macrophages, T-cell, NK cells	Mast cell SGN is substituted with heparin, macrophage, NK killer, T-cell SGN has CS (CS-A, CS-E) side chains	Kolset and Pejler, 2011; Roy et al., 2017
Endocan	50 kDa Endothelial cell DS cell surface PG also found circulating in bloodstream	DS chain binds *L-*, *P*-Selectin FN, chemokines, cytokines. RANTES, SDF-1*β*, IL-8, MCP-1, IFN-*γ*, PF-4, MK, PTN, FGF-2, FGF -7	Maurage et al., 2009; Kali and Shetty, 2014; Gaudet et al., 2020
Testican-1, 2, 3	Testican-1 and -2 are CS/HS PGs, of the BM-40/SPARC/osteonectin family. Testican 3 is a brain specific HS PG	Testican-1 is upregulated in neurons and astrocytes following brain injury. Testican 1-3 regulate MT_MMP and cathepsin L activity in neural tissues.	Marr et al., 2000; Iseki et al., 2011; Hartmann et al., 2013
CD 141	Thrombomodulin is a component of the endothelium, thrombin inhibitor	Inhibits thrombin in the endothelium protein C- anticoagulant system. Has anti-inflammatory barrier-stabilizing properties in ischemic stroke, enhancing vessel recovery and BBB repair.	
CD44	V3 splice variants bear CS chains	Binds Ezrin, fibrin/fibrinogen, FN, HA, OPN, Selectins-P,-E,-L.	Dzwonek and Wilczynski, 2015; Mooney et al., 2016
CD47	Neuron 50 KDa, 4 span TM CS-PG receptor	Neuroimmune regulatory protein, TSP-1 receptor, binds SIRPα. Regulates neuronal migration, proliferation and vascular cell survival, in innate and adaptive immunity, increases tissue resilience	Zhang H. et al., 2015; Matlung et al., 2017; Bedoui et al., 2018; Hutter et al., 2019; Li et al., 2021
Astrochondrin	Astrocyte cell surface PG	Binds laminin and type IV collagen in microvasculature and meninges.	Streit et al., 1993
Photomedin	Brain-specific glycoprotein of the eye neuroepithelium. member of the olfactomedin protein family	Photomedin interacts with CS-E to regulate axonal growth and differentiation of neural sensory epithelium	Furutani et al., 2005
FORSE-1 PG (forebrain-surface-embryonic)	LeX-substituted 286 kDa neuro-progenitor cell glycoprotein/PG	Lewis-X, SSEA-1 or CD15, Galβ(1-4)Fucα(1-3)GlcNAc-R oligosaccharide epitopes in FORSE-1 have roles in embryonic neural development	Gooi et al., 1981; Hakomori et al., 1981; Allendoerfer et al., 1995, 1999; Kelly et al., 2019

*ADAM-TS, a disintegrin and metalloproteinase with thrombospondin motifs; BBB, blood brain barrier; BM, basement membrane; BMP, bone morphogenic protein; CNS, central nervous system; CS, chondroitin sulfate; DS, dermatan sulfate; ECM, extracellular matrix; ERK, extracellular regulated kinase; FAK, focal adhesion kinase; FGF-2, fibroblast growth factor-2; HA, hyaluronic acid; HS, heparan sulfate; IL1, interleukin-1; LRR, leucine rich repeat; MMP, matrix metalloproteinase; MS, mass spectrometry; NCAM, neural cell adhesion molecule; PCM, pericellular matrix; PNS, peripheral nervous system; SLRP, small leucine repeat proteoglycan; SV, sub-ventricular; TLR4, Toll-like receptor-4; TM, transmembrane; TNF-α, tumor necrosis factor-α; MK, midkine; PTN, pleiotrophin; FN, fibronectin; TSP, thrombospondin; SGN, serglycin; ITI, inter-α-trypsin inhibitor; SVZ, sub-ventricular zone; WISP-1, Wnt1-inducible-signaling pathway protein 1, CCN4; SDF-1, stromal cell derived factor-1, CXCL12; MCP-1, monocyte chemoattractant protein-1, CCL2; RANTES, Regulated upon Activation, Normal T Cell Expressed and Presumably Secreted, chemokine ligand 5 (CCL5); PF-4, platelet factor-4; SSEA-1, stage specific embryonic antigen-1; DAMP, danger associated molecular pattern; SPARC, secreted protein acidic and rich in cysteine.*

### Roles for the CS-Rich Lectican PG Family in Perineuronal Net Structures

The lectican family of neural PGs have similar structures to aggrecan but do not contain keratan sulfate (KS) or a G2 globular domain. Furthermore, their molecular dimensions are smaller due to shorter core proteins and less extensive distributions of CS side-chains (Yamaguchi, 2000). Lectican PGs occur as diffuse ECM components and as dense PNN structures attached to HA through interactions with lectican N-terminal HA-binding regions. This aggregate is stabilized by tenascin-R and Bral-1 (Hyaluronan and Proteoglycan Link Protein 2; HAPLN2). The form of aggrecan found in brain differs from that of cartilage aggrecan in that it contains less KS chains, and its CS chains are less densely distributed along the CS1 and CS2 core protein regions (Hayes and Melrose, 2020a). Some CS chains in neural aggrecan are replaced by HNK-1 trisaccharide which also attaches to the same core protein linkage tetrasaccharide as CS. Once the HNK-1 trisaccharide is assembled chain elongation ceases resulting in a reduction in CS chain density but introduces cell interactive properties. Neural aggrecan guides neural crest progenitor cell migration during embryonic neurogenesis and formation of the neural tube and notochord (Hayes and Melrose, 2020a). Preclinical spinal cord injury (SCI) and traumatic brain injury (TBI) animal model studies demonstrate that the enzymatic degradation of CS-PGs from gliotic scars using chondroitinase ABC improves neuronal functional recovery (Bradbury and Carter, 2011; Cheng et al., 2015; Muir et al., 2019). Endogenous degradation of the core protein of CS-PGs by ADAMTS-4 also improves neuronal functional recovery (Tauchi et al., 2012). While the CS-A and CS-C side chains of the lecticans inhibit neural repair, not all CS-PGs inhibit axonal re-growth (Mencio et al., 2021). PGs containing over-sulfated CS-B, and CS-E promote neurite outgrowth and functional recovery (Bovolenta and Fernaud-Espinosa, 2000). The EGF-like motif in the G3 domain of the lecticans has also been shown to regulate cell migration and tissue repair (Aguirre et al., 2007; Du et al., 2010). Overexpression of human EGFR in CNP (hEGFR) mice accelerates remyelination and functional recovery following focal demyelination. Progenitor cells over-expressing NG2 PG also improve re-myelination through EGFR mediated cell signaling (Keirstead and Blakemore, 1999; Aguirre et al., 2007). PNNs surrounding the soma and dendrites of a number of neuronal cell types are prevalent during neural development and maturation (Carulli and Verhaagen, 2021). A similar structure, the perinodal ECM surrounds the axonal nodes of Ranvier and appear after re-myelination, acting as a protective ion-diffusion barrier (Bekku and Oohashi, 2019; Fawcett et al., 2019). Perinodal structures in the Nodes of Ranvier also contain PNN components such as brevican and versican V2 (Bekku et al., 2009; Dours-Zimmermann et al., 2009).

Perineuronal net are variably distributed in the brain, the somatosensory frontal lobes of the cerebral cortex have a particularly high density of PNNs, however, they are sparsely distributed in the sub-ventricular and sub-granular dentate gyrus of the hippocampus. These regions contain neuro-progenitor stem cell niches termed fractones (Mercier and Arikawa-Hirasawa, 2012; Sato et al., 2019). Abnormal PNN formation impacts on neural development and may result in degenerative synaptic pathology in schizophrenia (Pantazopoulos et al., 2021), bipolar disorder, major depression, and autism spectrum disorders (Sorg et al., 2016). CS-PGs in PNNs control synaptic plasticity, and have roles in memory in the aging brain, deterioration of PNNs contribute to the age-dependent decline in brain function. Recent work has revealed the importance of PNNs in the control of CNS plasticity. Digestion, blocking or removal of PNNs impedes functional recovery after a variety of CNS lesions. Deficient PNN numbers are implicated in a number of psychiatric disorders and suggested as therapeutic targets in their treatment (Dityatev et al., 2021). Incorrect assembly of PNNs or degradation of PNN components by excessive MMP activity can lead to the development of epilepsy (Rankin-Gee et al., 2015; Dubey et al., 2017; Mencio et al., 2021). Deficient levels of HA in PNN structures promote epilepsy and spontaneous convulsions in animal models (Perkins et al., 2017). The CS-PGs of PNNs have important functional roles to play in perisynaptic structures that prevent the development of Alzheimer’s disease (AD) (Morawski et al., 2012), cortical regions with abundant levels of ECM CS-PGs are less affected by degenerative features associated with the development of AD (Bruckner et al., 1999). PNNs also have important roles to play in Schizophrenia and Bipolar disorder (Berretta, 2012; Mauney et al., 2013). In unaffected individuals, the density of PNNs in the prefrontal cortex increases during pre-puberty and early adolescence. However, in patients with schizophrenia, a 70% reduction in PNN numbers in the prefrontal cortex has been observed (Mauney et al., 2013). The organization and function of PNNs is also disturbed in bipolar disorder (Gandal et al., 2018). Stem cells have been administered to promote recovery of normal PNN structure in an attempt to reverse these debilitating conditions (Forostyak et al., 2014). With an appreciation of PNNs and their important contributions to synaptic stability (Miyata et al., 2018), plasticity, memory and cognitive learning in normal brain tissues this has led to the identification of abnormalities in PNN assembly or expression of PNN components associated with particular neurodegerative conditions (Yamaguchi, 2000; Wen et al., 2018). Thus PNNs have become a therapeutic focus in the treatment of these conditions (Dityatev et al., 2006, 2010, 2021).

## Neural Proteoglycans

### Aggrecan

In the CNS/PNS, aggrecan core protein contains KS, HNK-1 trisaccharide and CS side chains (Hayes and Melrose, 2020a) that convey unique tissue-specific functional properties (Figure 3A). Aggrecan’s ability to form macro-aggregates with HA provides water imbibing, space-filling and matrix stabilizing properties to the PNS/CNS ECM and in brain establishes ionic gradients and microcompartments important for the optimal activity of neural cell populations.

The ability of the aggrecan core protein to assemble CS and KS chains at high density provides its well-known water-imbibing properties. Specific arrangements of GAG chains on aggrecan are functional determinant providing unique tissue context-dependent regulatory properties over neural cell populations. The aggrecan core protein KS and CS side chains and N-linked oligosaccharides all display neurite outgrowth-inhibitory activity (Hering et al., 2020). The cell mediatory properties of aggrecan’s GAGs thus convey diverse regulatory roles in tissue development and in neuroprotective matrix stabilization of PNNs. Variation in the sulfation position and density on the CS side chains can influence morphogen and growth factor binding relevant to tissue development (Reichsman et al., 1996; Nandini and Sugahara, 2006; Nadanaka et al., 2008; Whalen et al., 2013; Mizumoto et al., 2015).

### Versican

Versican is a large member of the lectican family (Yamaguchi, 2000) with a 400 kDa core protein modestly substituted with CS side chains (Figure 3B). Versican occurs as four alternatively spliced isoforms, VO, V1, V2, V3 (Yamaguchi, 2000). Versican was named after its versatile roles as a cell instructional and ECM organizational functional PG in tissue development, cell migration, adhesion, proliferation, and differentiation. Versican V1 promotes neuritogenesis (Wu et al., 2004). Versican interacts with HA through its G1 globular domain, C-type lectin G3 motifs interact with tenascin-R to stabilize HA-versican macro-aggregates (Bignami et al., 1993) and with HNK-1-substituted cell adhesion proteins (Bignami et al., 1993), HNK-1 glycolipids (Miura et al., 2001), and sulfated GAGs (Miura et al., 1999). Free G1, G3 versican domains released by proteases have regulatory properties in cell adhesion, proliferation, apoptosis, migration, angiogenesis, invasion, and metastasis. Versican G3 domain regulates neurite growth and synaptic transmission of hippocampal neurons by activating EGFR (Xiang et al., 2006). NgR2 interacts with versican G3 suppressing axonal plasticity (Bäumer et al., 2014) and has a dominant-negative effect on astrocytoma cell proliferation (Wu et al., 2001). An 80 kDa N-terminal matricryptic fragment of versican (versikine) generated by ADAMTS-4 (a disintegrin and metalloproteinase with thrombospondin motifs) cleavage acts as an alarmin in the innate immune response (Yamada et al., 2011). Interactions between myeloma stromal and myeloid cells generates versikine, a DAMP (damage-associated molecular pattern) that may facilitate immune sensing of myeloma tumors (Hope et al., 2016). Versikine also occurs during connective tissue remodeling during embryonic development (Nandadasa et al., 2014). Versican V2 is highly expressed in the adult brain (Schmalfeldt et al., 1998), promotes angiogenesis (Yang and Yee, 2013), and interactions with neurons (Horii-Hayashi et al., 2008). Versican V1 induces neural differentiation and neuritogenesis (Wu et al., 2004). Versican isoforms are differentially distributed in gliomas, medulloblastomas, schwannomas, neurofibromas, and meningiomas. Versican V2 is the major isoform found in gliomas. Versican V0 and V1 are found in all tissues, Versican V3 is found in all tissues except medulloblastomas.

### Neurocan

Neurocan has a widespread distribution in the CNS/PNS and is a component of PNNs (Schmidt et al., 2020) and regulates synaptic signaling (Sullivan et al., 2018). Neurocan (Figure 3C) has roles in neurodegenerative disorders (Lin et al., 2021). β-amyloid increases neurocan expression in astrocytes through Sox9 influencing the development of AD. Mutations in the neurocan gene predispose to bipolar disorder and schizophrenia (Mühleisen et al., 2012; Raum et al., 2015). Neurocan regulates neural migration and axonal development in the cerebral cortex influencing the folding of the occipital and pre-frontal lobes and an increased probability of developing schizophrenia (Schultz et al., 2014).

### Brevican

Brevican is the smallest lectican CS-PG family member (Figure 3D) present in PNNs in some cases, but aggrecan and versican are the principal lecticans in PNNs (Yamaguchi, 1996). Brain-enriched hyaluronan-binding protein (BEHAB) is an N-terminally cleaved (Matthews et al., 2000) bioactive fragment of brevican that is dramatically increased in human gliomas (Nutt et al., 2001; Viapiano et al., 2008) where it promotes glial cell motility and the aggressiveness of gliomas (Yamaguchi, 1996; Gary and Hockfield, 2000; Nutt et al., 2001; Viapiano et al., 2008; Giamanco and Matthews, 2020).

## Other Non-Lectican Large Neural Proteoglycans

### Phosphacan/Receptor Protein Tyrosine Phosphatase-Zeta (RPTP-ζ)

A cell membrane bound precursor form of phosphacan (RPTP-ζ) (Figures 4A,B) is processed by proteases to release a soluble PG ectodomain (Figure 4C) called phosphacan (Chow et al., 2008), truncated and non-GAG substituted forms of phosphacan have also been described (Figure 4D) with neurite outgrowth promoting activity (Fujikawa et al., 2017). This property is thus due to the core protein in some phosphacan species while neurite outgrowth activity may also be conveyed by GAG components, such as oversulfated CS-B and CS-E, in other phosphacan glycoforms (Dobbertin et al., 2003; Hikino et al., 2003). Phosphacan populations bearing KS and HNK-1 have also been described as well as the more common CS-glycanated form (Melrose, 2019b). Phosphacan promotes PNN formation (Eill et al., 2020). RPTP-ζphosphacan contain extracellular carbonic anhydrase (CAH) and fibronectin type III repeat domains, which foster protein–protein interactions (Milev et al., 1994; Lamprianou et al., 2011). A truncated 90 kDa phosphacan form is not a PG, but is substituted with the HNK-1 trisaccharide which facilitates interactive properties with a number of cell adhesion and ECM molecules (Garwood et al., 2003). Phosphacan promotes neuron–glial interactions, neuronal differentiation, myelination, and axonal repair. The CAH carbonic anhydrase domain of phosphacan promotes protein–protein recognition, induces cell adhesion, neurite outgrowth of primary neurons, and differentiation of neuroblastoma cells (Adamsky et al., 2001); contactin is a phosphacan neuronal receptor that regulates neural development and axonal repair.

### NG2 Proteoglycan/CSPG4

CSPG4 modular transmembrane CS-PG also occurs as a soluble protease generated form (Schäfer and Tegeder, 2018; Figure 4G). CSPG4 is expressed by oligodendrocyte precursor cells (OPCs), NG2 glia (Butt et al., 2019), pericytes (Girolamo et al., 2013), activated astrocytes in damaged neural tissues (Anderson et al., 2016) and fibroblasts and macrophages associated with the meninges (Tamburini et al., 2019). NG2/CSPG4 is the largest complex macromolecule of the neuron surfaceome (Tamburini et al., 2019). OPCs are sensitive to electrophysiological stimulation through synaptic interactions that induce cellular proliferation and tissue repair.

The 290 kDa ectodomain of CSPG4 is released from OPCs by ADAM10 (α-secretase) (Moransard et al., 2011; Clarke et al., 2012; Huang et al., 2014) and are a major source of neural CSPG4 (Jones et al., 2002). Neurons, astrocytes, and microglial cells do not express CSPG4. Glioblastoma cells (Moransard et al., 2011; Huang et al., 2014), endothelial cells and pericytes in gliotic scars express CSPG4 (Jones et al., 2002; McTigue et al., 2006). NG2 PG binds type V and VI collagen through its central non-globular domain (Clarke et al., 2012; Huang et al., 2014) and with integrins (Sakry and Trotter, 2016). C-terminal LamG domains of NG2 interact with BM components and are crucial for formation of synaptic neuroligin-neurexin complexes and glial cell signaling (Jeong et al., 2017) and also interact with matriglycan-dystroglycan, perlecan, agrin and type XVIII collagen to localize NG2PG in motor neuron endplates in the neuromuscular junction (NMJ) (Walimbe et al., 2020).

### Betaglycan

Betaglycan homo-dimeric transmembrane (TM) CS/HS PG (Mythreye and Blobe, 2009; Bilandzic and Stenvers, 2011) contains inhibin, FGF-2, Wnt, and TGF-β binding sites (Boyd et al., 1990; Segarini, 1991; Massagué et al., 1992; Sandbrink et al., 1996; Miyazono, 1997; Lewis et al., 2000; Gray et al., 2001; Kim et al., 2019; Bernard et al., 2020; Figure 5B). The HS chains of betaglycan bind FGF-2. Wnt signaling is regulated independently of TGF-β (Jenkins et al., 2018). HS inhibits Wnt signaling, while CS promotes Wnt signaling (Jenkins et al., 2016, 2018). Betaglycan *N*-and *O*- linked oligosaccharides and GAG chains, modulate betaglycan’s growth factor-mediated, vascular and cancer cell migratory properties (Pantazaka and Papadimitriou, 2014) and Inhibin A and B binding (Makanji et al., 2007). Fragments of betaglycan released by plasmin and MMPs act as circulating antagonists to normal betaglycan interactions. Inhibin/activin subunits and betaglycan are co-localized in the human brain (MacConell et al., 2002; Miller et al., 2012). Betaglycan-FGF-2 mediate neural proliferation and differentiation in neuroblastoma (Knelson et al., 2013). TGF-β also enhances glioma migration and invasion. TGF-β TbetaR I-III signaling phosphorylates Sma and MAD-related protein (SMAD), soluble TbetaR-I-III antagonize this process (Naumann et al., 2008). TGF-β enhances adult neurogenesis in the sub-ventricular zone (SVZ) and supports pro-neurogenic roles for TGF-β (Battista et al., 2006; Mathieu et al., 2010). Activins and inhibins, stimulate or inhibit secretion of FSH and the differentiation, proliferation and function of many cell types (Vale et al., 2004). Activin receptors highly expressed in neuronal cells, and activin mRNA are upregulated by neuronal activity. Models of TBI display enhanced activin A expression exacerbated by hypoxic/ischemic injury, mechanical irritation, and chemical damage (Florio et al., 2007). FGF-2 is neuroprotective and prevents apoptosis by strengthening anti-apoptotic pathways promoting neurogenesis in the adult hippocampus by upregulation of activin A activity (Woodruff, 1998; Alzheimer and Werner, 2002; Florio et al., 2007).

## The Small Neural Proteoglycans

### Neuroglycan C

Neuroglycan C is a part time (Oohira et al., 2004) brain specific TM (Watanabe et al., 1995; Yasuda et al., 1998; Shuo et al., 2004) 150 kDa CS-PG (Figure 4J) with a 120 kDa core protein that can also be shed by MMPs (Shuo et al., 2007), GAG-free forms of CSPG5 have also been described. Neuroglycan C, is a novel member of the neuregulin family (Kinugasa et al., 2004), interacting with pleiotropin (Nakanishi et al., 2010) producing neurite outgrowth-promoting activity mediated by phosphatidylinositol 3-kinase and protein kinase C (Nakanishi et al., 2006). CSPG5 forms peri-synaptic structures in the postnatal adult rat cortex (Jüttner et al., 2013). Impaired CSPG5 properties are evident in schizophrenia (So et al., 2010). Alternatively spliced forms have been identified in the human brain, recombinant CSPG5 induces phosphorylation of Erb2 and Erb3 and induces proliferation of neocorticol neurons (Kinugasa et al., 2004; Nakanishi et al., 2006). The neurite outgrowth promoting activity of neuroglycan C resides in its EGF and acidic amino acid domains (Nakanishi et al., 2006).

### Syndecans

Syndecan transmembrane HS/CS-PGs (Figures 4E,F) modulate cell adhesion, cell–cell interactions and ligand-receptor interactions that regulate neural plasticity, promote neural growth and development (Couchman et al., 2015; Gopal et al., 2021). Sdc-3 and Sdc-4 are found throughout the nervous system and have roles in motor neuron development (Liu et al., 2020), Slit/Robo signaling and guidance of axonal development (Steigemann et al., 2004). Sdc3 is a co-receptor for Heparin-Binding Growth-Associated Molecule (HB-GAM)/midkine-induced neurite outgrowth in perinatal rat brain neurons. HB-GAM acts as a local, synaptic factor that promotes presynaptic and postsynaptic differentiation during neural development. Sdc3 also has roles in adult neuronal synaptic plasticity in the hippocampus in rat models following injury and regulates the neuronal internodal axonal ECM during re-myelination in growth, remodeling and repair (Steigemann et al., 2004). Sdc3 and Sdc4 promote functional recovery of neural tissues re-organizing sodium and potassium channels (Steigemann et al., 2004). Oligodendrocytes are sensitive to electro-stimulation, and this maintains their membrane polarization required for the promotion of axonal repair processes. Sdc1 is upregulated by neurons following TBI and SCI (Murakami et al., 2015). Sdc1 and Sdc3 knockdown in dorsal root ganglia (DRG) neurons induces short neurite extensions suggesting roles in nerve regeneration, synaptic formation and plasticity (Akita et al., 2004; Steigemann et al., 2004). Syndecans shed from the cell surface by MMPs, act as soluble growth factor co-receptors that regulate cell migration acting antagonistically with cell surface syndecans competing for FGF and VEGF binding (Gopal et al., 2021) and interact with integrins potentially influencing cellular behavior, adhesion, spreading, migration, proliferation, tissue morphogenesis and pathogenetic tissue changes (Couchman et al., 2015).

### Decorin

Decorin (Figure 3E) regulates cellular survival, migratory, proliferative and angiogenic signaling and collagen fibril formation, sequesters TGF-β and antagonizes receptor tyrosine kinase family members, including EGFR and IGF-IR (Schönherr et al., 2005; Iozzo et al., 2011; Neill et al., 2012). MayDay, a ∼12 kDa N-terminal chemotactic factor, generated by macrophage-induced MMP-12 cleavage of decorin, recruits mesenchymal stem cells (MSCs) to damaged tissue regions *in vitro* and *in vivo*, promoting tissue repair (Dempsey et al., 2020). *In situ* hybridization (ISH) has localized decorin in areas of microvascular proliferation within gliomas and may be a therapeutic target in anti-angiogenic therapy (Patel et al., 2020) or approaches targeting TGF-β activity in tumors (Birch et al., 2020). Decorin protects neuronal tissue from the damaging effects of anti-oxidants and neuroinflammation following TBI by inactivation and has anti-tumor activity by inhibiting glioma cell migration (Yao et al., 2016). Decorin inhibits TGF-β activity, fibrous scar formation in neural tissues following trauma.

### Biglycan

Biglycan (Figure 3F) is synthesized by astrocytes (Koops et al., 1996) and immune cells (Mohan et al., 2010) and has neurotrophic activity, stimulates glial cell proliferation (Kikuchi et al., 2000) and neuronal cell survival (Koops et al., 1996). It is part of the proteome of the normal human retrobulbar optic nerve (Zhang et al., 2016) and is massively upregulated around gliotic scars following trauma (Stichel et al., 1995). NF-κB upregulates biglycan, protecting human neuroblastoma cells from nitric oxide (NO)-induced cell death by inhibiting AMPK-mTOR mediated autophagy and intracellular reactive oxygen species (ROS) production from mitochondrial oxidative bursts (Wang et al., 2015), targeting Erk1/2 and p38 signaling pathways to prevent NO-induced neuronal cell apoptosis (Chen et al., 2020). Biglycan regulates neuroinflammation (Xie et al., 2020) through M1 microglial cell activation in the early stages of subarachnoid hemorrhage, targeting Erk1/2 and p38 signaling pathways (Chen et al., 2018). Biglycan binds to Notch-3 and accumulates in cerebral autosomal dominant arteriopathy with subcortical infarcts and leukoencephalopathy (CADASIL) (Zhang X. et al., 2015). Transcriptomic profiling of the hypothalamus and hippocampus, supports a central regulatory role for biglycan (bgn) in molecular pathways linking metabolic events with the immune response, and neuronal plasticity (Ying et al., 2018). Transcriptomic profiling of hypothalamus, hippocampus, and liver supports regulatory roles for Bgn in molecular pathways involved in metabolism, the immune response, and neuronal plasticity (Ying et al., 2018).

### Epiphycan

The synaptic poles of inner hair cells of the cochlea have audio-sensory properties and are surrounded by basket-like ECM structures with similar roles to the PNNs of neurons in the CNS (Sonntag et al., 2015). Epiphycan (Figure 3G) and aggrecan are cochlear components and of the gel-filled tectorial membrane which detects auditory signals and transmits these to sensory hair cells (Melrose, 2019a). Epiphycan is expressed by cochlear supporting cells and is necessary for normal hearing. Epiphycan mRNA is abundantly expressed in the cochlea in the organ of Corti of neonatal and adult mice. The cochlea of epiphycan knockout (KO) mice display a normal morphology, however, the auditory brain-stem response is altered since epiphycan is necessary for normal auditory function (Hanada et al., 2017). These PNN like structures surround high function neuron types which respond to signals received from inner sensory hair cells, transducing audio signals into mechanical stimuli and receptor-mediated action potentials which are sent to spiral ganglion neurons (Sonntag et al., 2015). These neuron types operate at very high discharge rates and efficiently convey signals to the auditory brainstem for further processing. The hearing loss evident in cartilage matrix deficiency (CMD) mice is related to aggrecan deficiency in the cochlea (Melrose, 2019a).

### Appican

Two variants of the related amyloid precursor-like protein 2 (APLP2) carry single CS-E side chains, which bind midkine and pleiotrophin (Thinakaran and Sisodia, 1994; Shioi et al., 1995; Thinakaran et al., 1995). Multiple splice variants of amyloid-beta precursor protein (APP) (Figure 4I) and APLP2 arise from alternative splicing of three exons in APP and two exons in APLP2 (Kitaguchi et al., 1988; Ponte et al., 1988). The CS attachment site on APP/APLP2 is located adjacent to the membrane-spanning domain through deletion of 18 (APP) or 12 (APLP2) amino acids (Thinakaran et al., 1995). Splice variants also occur lacking CS side chains. APP and APLP are widely distributed in the CNS/PNS, APP is expressed by glial cells in the CNS/PNS.

### Bikunin/ITI

Bikunin (inter-α-trypsin inhibitor light chain) is synthesized by neurons (Chen et al., 2016), occurring as a tissue form and small circulating PG containing a single CS chain (Figure 3J). Bikunin displays anti-inflammatory, anti-protease, anti-microbial, anti-viral properties and also functions as a growth factor (Fries and Blom, 2000; Lord et al., 2020). Bikunin is expressed in brain tissue (Takano et al., 1999; Kim et al., 2020) and accumulates in brain tumors. Bikunin CS chains contain embedded disulfated CS-D motifs (Lord et al., 2013, 2020). A related Kunitz protease inhibitor, placental bikunin (hepatocyte growth factor activator inhibitor type-2) has been reported to inhibit glioblastoma tumor invasion (Hamasuna et al., 2001), however, this is a dissimilar protein to serum bikunin. Traumatic impact to the brain and spinal cord can release nuclear components such as histone H1 into the circulation or cerebrospinal fluid (CSF). Histone H1 has neuro-stimulatory effects and activates the innate immune response in the CNS mediated by microglial cells (Gilthorpe et al., 2013). This promotes neural cell survival, up-regulates major histocompatibility complex (MHC) class II antigen expression and is a powerful microglial chemoattractant. Release of histone H1 from the degenerative CNS drives a positive immune response (Gilthorpe et al., 2013) but can also be cytotoxic. Plasma immune tolerance induction (ITI) neutralizes the cytotoxic effects of histone H1, decreasing histone-induced platelet aggregation (Chaaban et al., 2015) through complexation of the histone with the negatively charged CS GAG chains of ITI (Chaaban et al., 2015). Hypoxic-ischemic encephalopathy predisposes infants to long-term cognitive decline impacting on life quality and healthcare resources (Chen et al., 2019). ITI regulates neonatal inflammation, decreases damage to brain tissues (Chen et al., 2019) and neuronal cell death, attenuates glial responses and leucocyte invasion with long-term beneficial effects in neonatal models of brain injury (Koehn et al., 2020).

### Serglycin

Serglycin (Figure 3H) is a small intracellular PG present in secretory granules of hemopoietic and endothelial cells (Kolset and Tveit, 2008) with regulatory properties over immune cells (Kolset and Pejler, 2011). It also promotes the development and aggressiveness of many tumor types including glioblastoma and is a glioblastoma biomarker (Roy et al., 2017; Manou et al., 2020). Suppression of serglycin in LN-18 shSRGN mutant cells results in retarded glioma proliferation, migration and invasive potential (Manou et al., 2020). Serglycin expression is elevated in astrocyte-glioma co-cultures. Astrocytes promote glioblastoma growth and is a potential glioma therapeutic target (Mega et al., 2020).

### Endocan

Endocan (Figures 3L, 5C) is a small endothelial cell surface DS-PG found in cerebral blood vessels and is a small circulating PG in the blood stream (Frahm et al., 2013). Human umbilical vein endothelial cells (HUVECs) produce a truncated, alternatively spliced form of endocan which is neither glycosylated or secreted (Tsai et al., 2002). Circulating PGs are relatively rare; examples include endocan, bikunin, and macrophage colony stimulating factor-1 (Aitkenhead et al., 2002; Zhao et al., 2004). Endocan shares no homologies with other ECM PGs (De Freitas and Lassalle, 2015), does not contain LRRs or C-type lectin domains. Endocan, endothelial cell specific molecule-1 (ESM-1) encoded by the ESM-1 gene is an atypical DS-PG, with a single DS chain and distinctive structural and functional properties (Xing et al., 2016; Sun et al., 2019). Endocan is expressed by endothelial cells, regulated by proinflammatory pro-angiogenic molecules, has matrix-binding properties and is a marker of endothelial cell activation. TNF-*α*, IL-1, TGF-*β*1, FGF-2, and VEGF-2 induce endocan expression *in vitro*, IFN-*γ* inhibits TNF-*α* induced upregulation of endocan (Scherpereel et al., 2003). Endocan is associated with neuroinflamation in highly vascularized tumors in meningiomas, gliomas and lung cancer (Maurage et al., 2009) and with new blood vessel development in glioma (Maurage et al., 2009), pituitary adenoma, renal cell carcinoma, pediatric brain injury (Lele et al., 2019) and is a biomarker of cerebral damage (Morleo et al., 2019). Endocan expression is upregulated in human cytomegaloviral infection which increases glioma development in brain tissues (Scherpereel et al., 2003; Xing et al., 2016) leading to its suggestion as a therapeutic target in glioma (Atukeren et al., 2016). Endocan binds to lymphocytes and monocytes through high affinity interactions with integrin CD11a/CD18 lymphocyte function associated antigen-1 (LFA-1). A protease cleaved form of endocan (p14) antagonizes these interactions (Gaudet et al., 2020). Endocan promotes adhesion of monocytes and endothelial cells (Sun et al., 2019). The DS chains of endocan bind and activate hepatocyte growth factor (HGF) *in vitro* (Lyon et al., 1998; Mythreye and Blobe, 2009), L- and *P*-Selectins, fibronectin, chemokines, cytokines, RANTES, Stromal Cell-Derived Factor-1*β* (SDF-1*β)*, IL-8, monocyte chemoattractant protein-1 (MCP-1), IFN-*γ*, and platelet factor-4 (PF-4), midkine, pleiotrophin, FGF-2, and FGF-7 (Sarrazin et al., 2006).

### Testican

Testican-1 and -2 are CS/HS PGs (Figure 3K), of the BM-40/SPARC/osteonectin family of extracellular calcium-binding proteins consisting of a signal peptide, a follistatin-like domain, a central Ca2+-binding domain, a thyroglobulin-like domain, and a C-terminal GAG attachment region. Testican-1 and 2 are expressed by multiple neuronal cell types in olfactory bulb, cerebral cortex, thalamus, hippocampus, cerebellum, and medulla (Marr et al., 2000). Neuronal testican-1 is upregulated following brain trauma (Iseki et al., 2011) and is also expressed by activated astrocytes. Testican-1 modulates neuronal attachment and MMP activation (Bocock et al., 2003; Edgell et al., 2004), inhibits membrane type MMPs and cathepsin-L but not cathepsin-B. Testican-1 contains a single thyropin domain highly homologous to domains in cysteine proteinase inhibitors, the CS chains of testican-1 are essential for inhibition of cathepsin-L. Testican-2 is a HS/CS PG, has GAG-substituted and GAG-free forms that inhibit neurite extension, regulating neuronal growth and development (Schnepp et al., 2005). Testican-2 abrogates the inhibition of metallothionein-1(MT1)-MMP- or MT3-MMP-mediated pro-MMP-2 activation by testican-1 (Nakada et al., 2003). Testican-3, a HS-PG exclusive to brain tissues suppresses MT1-MMP mediated activation of MMP-2 and tumor invasion (Hartmann et al., 2013).

### Thrombomodulin (CD141)

Thrombomodulin (Figure 4H) inhibits thrombin as part of the anticoagulant protein C-system in the endothelium, is anti-inflammatory and promotes barrier-stabilization. Thrombomodulin is a protective factor in the brain during ischemic stroke, enhancing vessel post-ischemic recovery in the blood brain barrier. Thrombin’s physiological roles in the brain stabilize normal brain function in synaptic transmission and plasticity through direct or indirect activation of Protease-Activated Receptor-1 (PAR1) and has neuroprotective roles in neurological diseases (Krenzlin et al., 2016).

### Cluster of Differentiation 44

Cluster of differentiation 44 (CD44), a major transmembrane glycoprotein HA receptor (Figure 4K) in the CNS/PNS, has roles in cell division, migration, adhesion, and cell–cell and cell–ECM signaling (Dzwonek and Wilczynski, 2015). Alternatively spliced CD44v3 is a CS-PG, 20 isoforms of CD44 have been reported associated with several kinds of tumors. CD44 expression is highly dynamic and transitions between different isoforms during tumor development (Lah et al., 2020). In glioma, CD44 and integrins attach the cell to ECM forming focal adhesion complexes and generate traction forces that facilitate cell spreading, essential in the cell migratory machinery in glioma cell invasion (Mooney et al., 2016). In the normal brain, CD44 is a major HA receptor that interacts with osteopontin, collagens, and MMPs stabilisating and remodeling the CNS/PNS ECM (Dzwonek and Wilczynski, 2015). HA is highly interactive through CD44, conveying cell instructional cues, and ECM stabilization, hydration and space-filling properties thus maintaining tissue compartmentalisation, ionic gradients and niches important in the metabolism of neural cell populations (Sherman et al., 2015; Peters and Sherman, 2020), including neural progenitor stem cell niches (Preston and Sherman, 2011).

### Cluster of Differentiation 47

Cluster of differentiation 47 (CD47) (Figure 5A), originally named integrin-associated protein (IAP) is a receptor for thrombospondin-1 (TSP-1) regulates cellular migration, proliferation, and the survival of vascular cells, in innate and adaptive immune regulation (Barclay and Van den Berg, 2014; Murata et al., 2014). TSP-1 acts via CD47 to inhibit NO signaling in the vascular system supporting blood pressure by regulation limiting endothelial nitric oxide synthase (eNOS) activation and endothelial-dependent vasorelaxation. CD47 is a ligand for signal regulatory protein α (SIRPα), also known as SHPS-1/BIT/CD172a). The CD47-SIRPα signaling system is a cell-cell communication system (Zhang H. et al., 2015; Matlung et al., 2017; Weiskopf, 2017). CD47-SIRPα interactions have been termed an innate immune checkpoint in macrophages (Li et al., 2021). Blockade of anti-phagocytic CD47-SIRPα interactions using humanized antibodies to CD47 (Hu5F9-G4) has yielded promising results in preclinical studies of a number of human malignancies including pediatric brain tumors: medulloblastoma, atypical teratoid rhabdoid tumors, primitive neuroectodermal tumor, pediatric glioblastoma, and diffuse intrinsic pontine glioma (Gholamin et al., 2017) and accelerates the clearance of hematomas in experimental intraventricular hemorrhage (Ye et al., 2021). Thus by targeting the immunological checkpoint complex CD47-SIRPα, the development of glioblastoma can be inhibited, the function of phagocytic, dendritic and T-lymphocytes enhanced and the efficiency of tumor cell removal improved by innate and adaptive immune responses (Hutter et al., 2019; Hu et al., 2020; Kuo et al., 2020; Zhang et al., 2020).

### Astrochondrin

Some specialized CNS proteins such as astrochondrin, a cell surface CS-PG of astrocytes carry L2/HNK-1 and L5 carbohydrate structures interactive with ECM components such as laminin and type IV collagen this may facilitate interaction of astrocyte foot processes with the brain microvasculature and meningeal membranes (Streit et al., 1993).

### Photomedin

Photomedin is another brain-specific glycoprotein of the eye neuroepithelium that interacts with CS-E (Furutani et al., 2005). Photomedin is a member of the olfactomedin protein family and has regulatory roles in axonal growth and differentiation of sensory cilia in the neural epithelium.

### FORSE-1 (Forebrain-Surface-Embryonic) Proteoglycan

FORSE-1 contains LeX-carbohydrate, stage specific embryonic antigen-1 (SSEA-1) or CD15, terminal Galβ(1-4)Fucα(1-3)GlcNAc-R oligosaccharide epitopes with roles in embryonic neural development (Allendoerfer et al., 1995, 1999; Kelly et al., 2019).

## CS-PGs Regulation of Neuronal Cell Signaling

**FIGURE 6:**
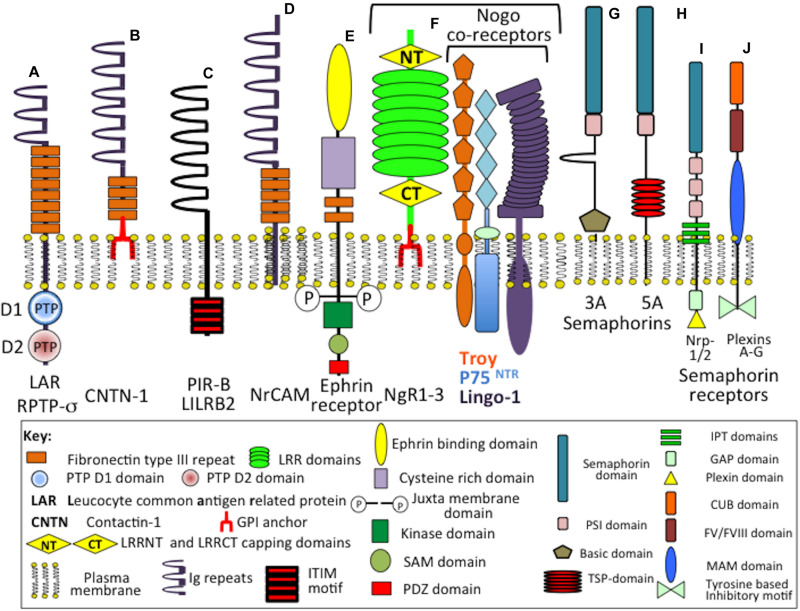
Schematic depiction of the modular structures of diverse cell surface molecules expressed by neural cells that bind CS-proteoglycans. The fibronectin-III and Ig repeat transmembrane protein LAR (Leukocyte common antigen-related receptor protein tyrosine phosphatase **(A)** and homologous contactin-1 (CNTN-1) **(B)** Murine paired immunoglobulin receptor B (PirB) and its human ortholog leukocyte immunoglobulin-like receptor B2 (LILRB2) **(C)**, neural cell adhesion molecule (NrCAM) **(D)**. The ephrin receptor **(E)**, Nogo and its co-receptors **(F)**, semaphorins 3A **(G)**, 5A **(H)** and the neuropilin **(I)** and neuroplexin receptors **(J)**.

**FIGURE 7:**
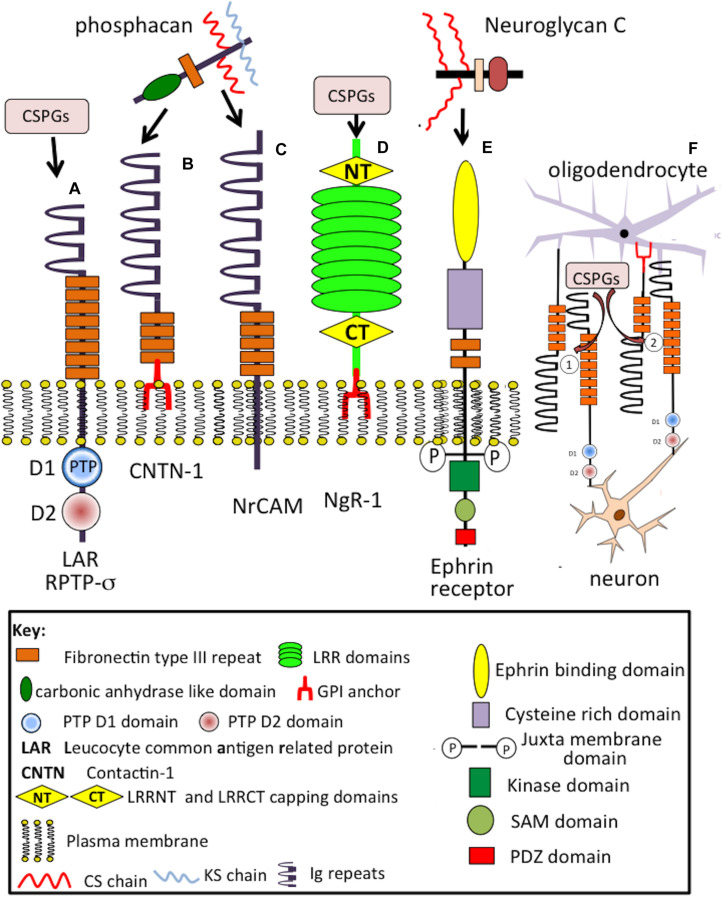
Schematic representation of the structural organization of neural cell adhesion molecules that act as CS-PG receptors **(A–C)**, and the neural NgR1 **(D)** and ephrin receptors **(E)** where binding of CS-PGs results in an inhibitory signal to the neuron. Ephrin acts as a receptor for Neuroglycan-C, while phosphacan binds to Contactin-1 **(B)** and NrCAM **(C)**. LAR acts as a receptor for several CS-PGs. Oligodendrocyte-neuronal communication mediated by LAR-NrCAM (Condomitti and de Wit, 2018) or LAR-contactin-1 interactions (Zhang P. et al., 2018) can be regulated by competitive binding of CS-PGs to these cell surface components **(F)**.

Neural cell populations including astrocytes, oligodendrocytes, neurons, endothelial cells and pericytes of the brain microvasculature and microglial cells synthesize a range of CS-PGs that interact with a variety of cell surface molecules and receptors (Figure 6). These modulate cellular processes that control CNS/PNS function and repair (Djerbal et al., 2017; Figure 7). CS-PGs contribute to the structural integrity and compartmentalisation of the brain ECM and have important organizational functional roles in brain regions (Dityatev et al., 2010). CS-PGs operate at multiple functional levels involving interactions with growth factors, receptors, adhesion molecules, neural guidance proteins and ECM proteins (Djerbal et al., 2017). Transmembrane CS-PGs are active during cell-cell crosstalk, they may also be secreted or released from the cell surface by proteases to act remotely from their cells of origin and may antagonize normal transmembrane PG interactions (Figure 7F). CS-PGs are not uniformly distributed in the CNS/PNS but occur concentrated in neural growth cones and PNNs strategically positioned to control processes occurring at the cell-tissue interface (Shimbo et al., 2013; Sugitani et al., 2020). Growth cone receptor protein tyrosine phosphatases (RPTPs) bind with high affinity to CS-PGs, this controls axonal growth and provides guidance cues during regeneration, plasticity and neuronal development and in repair responses (Djerbal et al., 2017). CS-PGs attached to RPTP members can also exert repulsive guidance cues and inhibit neuritogenesis (Figure 6A). Lectican PG family members (neurocan, brevican, versican, aggrecan) are diffusely distributed in the CNS ECM and are also components of the denser PNNs. The CS component of CS-PGs vary in composition with tissue development. During embryonic development CS-C is a predominant isoform while CS-A is more abundant in adult neural tissues (Figure 2). The sulphation patterns and charge density are important functional determinants of CS glycoforms. The more highly charged CS-E, B and D are components of PNN PGs, CS-A and CS-C are diffusely distributed in the ECM distant from PNN structures (Djerbal et al., 2017). The chemo-repellent semaphorin 3A (SEMA3A), a component of PNNs (Battistini and Tamagnone, 2016; Fard and Tamagnone, 2020), interacts with CS-E and B but not CS-D, thus such interactions are not purely mediated by charge; saccharide sequences in CS chains also determine the interactive properties of CS-PGs and their core proteins also contain ECM interactive modules. Selective binding of midkine (MK) and brain-derived neurotrophic factor (BDNF) to CS-E of some CS-PGs leads to neurite outgrowth. The Nogo receptors NgR1 and NgR3 bind to Nogo and inhibit neurite outgrowth. NgR1 and NgR3 also bind specifically to CS-B, CS-D, and CS-E with high affinity, and this inhibits neurite outgrowth. Cell adhesion molecules are operative in cell–cell and cell–ECM interactions that regulate tissue integrity, cellular communication and cellular migration during CNS development and repair following trauma and are evident as pathological functional changes in neurological disorders. Neural cell adhesion molecule (NCAM) in particular, has specific roles in the promotion of neurite outgrowth of motor neurons that improves locomotor functional recovery following SCI. NCAM and neuroglia cell adhesion molecule (NgCAM) bind with high affinity to the CS-PG phosphacan, reducing neurite outgrowth and adhesion. Chondroitinase ABC moderately reduces phosphacan-NCAM binding showing this interaction is mainly mediated through phosphacan core protein interactions (Figure 7). Neurocan binding to NCAM and NgCAM also inhibits neurite outgrowth but unlike phosphacan, chondroitinase ABC abrogates this, showing that neurocan-NCAM interactions are mediated through CS. NCAM and NgCAM act as receptors for phosphacan and neurocan. Contactin-1 is a further GPI anchored cell adhesion molecule (CAM) that facilitates axonal growth and dendritic interactions that promote neurogenesis, CS-E binds contactin-1 with significant affinity and promotes neural growth. Thus, CS-PG binding to contactin-1 can modulate contactin-1 interactions that normally mediate cell-cell interaction (Figure 7F). CS-PG5 (neuroglycan C) forms peri-synaptic matrix assemblies that regulate neuronal synaptic activity in the cerebral cortex of rats (Pintér et al., 2020).

The semaphorins are a family of guidance proteins of embryonic peripheral nerve projections and have roles in synaptogenesis and the maintenance of neural interconnections in adulthood thus maintaining cerebral homeostasis. SEMA3A is upregulated following CNS injury, causes growth cone collapse by signaling through synaptic neuropilin-1 (Nrp-1) and plexin receptors and is also known as collapsin-1. SEMA3A in PNNs can modulate synaptic morphology and function (Goshima et al., 2002; Mecollari et al., 2014; Battistini and Tamagnone, 2016; Alto and Terman, 2017; Fard and Tamagnone, 2020). SEMA3A interacts with CS-E with high affinity and neuropilin-1 (Nrp-1) in SEMA3A-Nrp-plexin signaling complexes to potently inhibit neural sprouting following SCI, and also inhibits neural growth factor (NGF) (Mecollari et al., 2014; Fard and Tamagnone, 2020) (Figure 6). Compared to SEMA3A, SEMA5A is less well studied, but also has important functional roles in CNS development and response to injury (Conrad et al., 2010). SEMA5A contains a cluster of thrombospondin (TSP) repeats which promote neural outgrowth. CS-PGs interact with this TSP repeat region producing a neuro-repulsive response whereas HS-PGs produce a neuro-attractive response. Thus, CS has important SEMA5A regulatory properties. A proteomic surface plasmon resonance and microarray study by Conrad et al. (2010), showed that interactions with neurotrophic factors was not confined to the highly charged CS-E glycoforms and that significant interactions also occurred between CS-A and Sema 3E, Sema 6B and ephrin A3 (Conrad et al., 2010).

## Conclusion

This chapter has shown the impressive diversity in CS-PG form and function in the CNS/PNS. CS-PGs undertake many essential roles in neural tissues through the provision of a functional ECM for the many different cell populations resident in the CNS/PNS. The intricacies of some of the cell regulatory properties conveyed by CS-PGs have been illustrated, as has the complex interplay between distinct neural cell populations in the maintenance of CNS/PNS tissue function and homeostasis. Aberrations in the assembly of the ECM through processing defects in component CS-PGs can have serious functional consequences in brain tissues and can lead to neurodegenerative diseases. Such defects underline the fundamental importance of the ECM in normal tissue function and the potential of CS-PGs as promising therapeutic targets for future treatment of many of these neurodegenerative conditions.

## Author Contributions

JM obtained the funding, conceptualized the study, and wrote the initial draft of the manuscript. AH assisted in manuscript writing and editing and had intellectual input into data interpretation. Both authors approved the final version of the manuscript.

## Conflict of Interest

The authors declare that the research was conducted in the absence of any commercial or financial relationships that could be construed as a potential conflict of interest.

## Publisher’s Note

All claims expressed in this article are solely those of the authors and do not necessarily represent those of their affiliated organizations, or those of the publisher, the editors and the reviewers. Any product that may be evaluated in this article, or claim that may be made by its manufacturer, is not guaranteed or endorsed by the publisher.

## Footnotes

1
http://flybase.bio.indiana.edu

2
www.ncbi.nlm.nih.gov

3
www.ebi.ac.uk/embl/index.html

4
www.ddbj.nig.ac.jp

## Glossary

AD: Alzheimer’s disease
ADAM: A disintegrin and metalloproteinase domain-containing protein
ADAMTSL: A disintegrin and metalloproteinase with thrombospondin motifs
AMPK: 5′ adenosine monophosphate-activated protein kinase
APLP2: amyloid precursor-like protein 2
APP: amyloid-beta precursor protein
BBB: blood brain barrier
BDNF: brain-derived neurotrophic factor
BEHAB: brain-enriched hyaluronan-binding protein
Bgn: biglycan
BMP: bone morphogenic protein
C4ST-1: chondroitin 4-*O*-sulfotransferase-1
C4ST-2: Chondroitin 4-*O*-sulfotransferase-2
CADASIL: cerebral autosomal dominant arteriopathy with subcortical infarcts and leukoencephalopathy
CAM: cell adhesion molecule
CD44: cluster of differentiation 44
CD47: cluster of differentiation 47
Chn: chondroitin
CMD: cartilage matrix deficiency
CNP: 2′,3′-cyclic nucleotide 3′-phosphodiesterase
CNS: central nervous system
CPG: chondroitin proteoglycans
CS: chondroitin sulfate
CSF: cerebrospinal fluid
CS-PG: chondroitin sulfate proteoglycan
D/iD: GlcA/IdoA(2-*O*-sulfate)-GalNAc(6-*O*-sulfate)
D4ST-1: dermatan 4-*O*-sulfotransferase-1
DAMP: damage-associated molecular pattern
DCC: deleted in colorectal carcinoma
DDBJ: DNA Data Bank of Japan
Dlp: Dally-like protein
Dome: domeless
Dpp: decapentaplegic, a ligand of the TGF β superfamily
DRG: dorsal root ganglion/ganglia
DS: dermatan sulfate
DS-PG: dermatan sulfate proteoglycan
E/iE: GlcA/IdoA-GalNAc (4,6-*O*-disulfate)
ECM: extracellular matrix
EGF(R): epidermal growth factor (Receptor)
eNOS: endothelial nitric oxide synthase
ESM-1: endothelial cell specific molecule-1
FGF: fibroblast growth factor
FORSE-1: forebrain surface embryonic-1
FSH: follicle-stimulating hormone
GPI: glycophosphatidylinositol
HA: hyaluronic acid
HAPLN2: hyaluronan and proteoglycan link protein 2
HB-GAM: heparin-binding growth-associated molecule
hEGFR: human epidermal growth factor receptor
HGF: hepatocyte growth factor
Hh: Hedgehog
HNK-1: human natural killer-1
HS: heparan sulfate
HS-PG: heparan sulfate proteoglycan
HSPG2: heparan sulfate proteoglycan 2 (perlecan)
Hst: heparan sulfate sulfotransferase
HUVECs: human umbilical vein endothelial cells
iA: 4-sulfated IdoA-GalNAc
IAP: integrin-associated protein
iB: 2-sulfated IdoA-GalNAc
IFN-*γ*: interferon-*γ*
IGF-IR: insulin-like growth factor-1 receptor
IL: interleukin
ISH: *in situ* hybridization
ITI: immune tolerance induction
JAK/STAT: Janus kinase/signal transducer and activator of transcription
KO: knockout
Kon: Kon-tiki (NG2 homolog)
KS: keratan sulfate
LFA-1: lymphocyte function associated antigen-1
LRR: leucine-rich repeat
MADD: muscle arm development defective
MAP: mitogen-activated protein
MCAF: monocyte chemotactic and activating factor
MCP-1: monocyte chemoattractant protein-1
MHC: major histocompatibility complex
MIP: macrophage inflammatory protein
MK: midkine
MMP: matrix metalloproteinase
MSC: mesenchymal stem cell
MT-1: metallothionein-1
mTOR: mechanistic target of rapamycin
NCAM: neural cell adhesion molecule
NF- κ B: nuclear factor Kappa-light-chain-enhancer of activated B cells
NG2: neuron-glial antigen 2
NgCAM: neuroglia cell adhesion molecule
NGF: neural growth factor
NgR2: Nogo-66 Receptor Homolog2
NLG: neuroligin
NMJ: neuromuscular junction
NO: nitric oxide
Nrp-1: neuropilin 1
OA: osteoarthritis
OPC: oligodendrocyte precursor cells
PAP: 3′-phosphoadenosine 5′-phosphosulfate
PAPS: 3′-phosphoadenosine 5′-phosphosulfate
PAR1: protease-activated receptor-1, PG, proteoglycan
PNN: perineuronal net
PNS: peripheral nervous system
Pros: prospero
RANTES: regulated upon activation, normal T cell expressed and secreted
RNAi: ribonucleic acid interference
ROS: reactive oxygen species
RPTPs: receptor protein tyrosine phosphatases
RPTP- ζ: receptor protein tyrosine phosphatase- ζ
SCI: spinal cord injury
Sdc: syndecan
SDF-1β: stromal cell-derived factor-1β
SDN-1: syndecan homolog found in *Caenorhabditis elegans;*
SEMA3A: semaphorin 3A
SEMA5A: semaphorin 5A
SIRPα: signal regulatory protein α
SMAD: Sma and MAD-related protein
SOX9: SRY-box transcription factor 9
Sqv: squashed vulva gene
SSEA-1: stage specific embryonic antigen-1
SVZ: subventricular zone
TBI: traumatic brain injury
TGF-β: transforming growth factor- β
TM: transmembrane
TNFR: tumor necrosis factor receptor
TNF-α: tumor necrosis factor-α
TSP-1: thrombospondin-1
UDP: uridine diphosphate
UNC: uncoordinated
UST: uronyl 2-sulfotransferase
VEGF: vascular endothelial growth factor
Wdp: windpipe
Wg: wingless.
